# Evolution of Fluconazole-Resistant Candida albicans Strains by Drug-Induced Mating Competence and Parasexual Recombination

**DOI:** 10.1128/mBio.02740-18

**Published:** 2019-02-05

**Authors:** Christina Popp, Bernardo Ramírez-Zavala, Sonja Schwanfelder, Ines Krüger, Joachim Morschhäuser

**Affiliations:** a Institut für Molekulare Infektionsbiologie, Universität Würzburg, Würzburg, Germany; Tel Aviv University

**Keywords:** *Candida albicans*, drug resistance evolution, mating, parasexual recombination

## Abstract

The clonal population structure of Candida albicans suggests that (para)sexual recombination does not play an important role in the lifestyle of this opportunistic fungal pathogen, an assumption that is strengthened by the fact that most *C. albicans* strains are heterozygous at the mating type locus (*MTL*) and therefore mating-incompetent. On the other hand, mating might occur within clonal populations and allow the combination of advantageous traits that were acquired by individual cells to adapt to adverse conditions. We have investigated if parasexual recombination may be involved in the evolution of highly drug-resistant strains exhibiting multiple resistance mechanisms against fluconazole, an antifungal drug that is commonly used to treat infections by *C. albicans*. Growth of strains that were heterozygous for *MTL* and different fluconazole resistance mutations in the presence of the drug resulted in the emergence of derivatives that had become homozygous for the mutated allele and the mating type locus and exhibited increased drug resistance. When *MTL***a**/**a** and *MTL*α/α cells of these strains were mixed in all possible combinations, we could isolate mating products containing the genetic material from both parents. The initial mating products did not exhibit higher drug resistance than their parental strains, but further propagation under selective pressure resulted in the loss of the wild-type alleles and increased fluconazole resistance. Therefore, fluconazole treatment not only selects for resistance mutations but also promotes genomic alterations that confer mating competence, which allows cells in an originally clonal population to exchange individually acquired resistance mechanisms and generate highly drug-resistant progeny.

## Abstract (precis)

Sexual reproduction is an important mechanism in the evolution of species, since it allows the combination of advantageous traits of individual members in a population. The pathogenic yeast Candida albicans is a diploid organism that normally propagates in a clonal fashion, because heterozygosity at the mating type locus (*MTL*) inhibits mating between cells. Here we show that *C. albicans* cells that have acquired drug resistance mutations during treatment with the commonly used antifungal agent fluconazole rapidly develop further increased resistance by genome rearrangements that result in simultaneous loss of heterozygosity for the mutated allele and the mating type locus. This enables the drug-resistant cells of a population to switch to the mating-competent opaque morphology and mate with each other to combine different individually acquired resistance mechanisms. The tetraploid mating products reassort their merged genomes and, under selective pressure by the drug, generate highly resistant progeny that have retained the advantageous mutated alleles. Parasexual propagation, promoted by stress-induced genome rearrangements that result in the acquisition of mating competence in cells with adaptive mutations, may therefore be an important mechanism in the evolution of *C. albicans* populations.

## INTRODUCTION

The opportunistic fungal pathogen Candida albicans was thought to be an obligately diploid, asexual organism until its genome sequence revealed that it possesses a mating type-like locus (*MTL*) that is similar to the mating type loci of fungi with a known sexual cycle (1–3). Most *C. albicans* strains are *MTL*-heterozygous and contain both *MTL***a** and *MTL*α alleles, which prevents them from mating (4–6). However, genome rearrangements, including transient aneuploidies, are relatively frequent in *C. albicans* (7, 8), and mitotic recombination or whole-chromosome loss/duplication can result in *MTL* homozygosity (9–12). Such cells can switch to the mating-competent opaque cell morphology and are then able to mate with opaque cells of the opposite mating type (5, 13). The tetraploid mating products are unstable, and although meiosis has not been observed in *C. albicans* so far, they can lose chromosomes in a random fashion and eventually return to the diploid state to produce recombinant progeny containing genetic material from both parents (2, 3, 13–17). These events are referred to as a parasexual cycle, to distinguish it from a true sexual cycle that involves meiosis.

For mating to be beneficial, it should generate offspring with a new combination of traits that confers a selective advantage in relevant host niches, i.e., it should occur between genetically different cells. However, individual humans are usually colonized with their own specific *C. albicans* strain (18–20), which reduces the chances of an encounter between unrelated strains. Even when several strains reside in the same host niche, the facts that most *C. albicans* strains are *MTL*-heterozygous and that sporadic *MTL*-homozygous cells first have to switch to the opaque phase to become mating-competent strongly limit the opportunity of mating between them. Indeed, population-genetic analyses have shown that the population structure of *C. albicans* is primarily clonal, indicating that mating is rare in this fungus (21, 22).

On the other hand, mating may actually be more frequent in *C. albicans* than supposed from the above-mentioned limitations if it occurs between cells within a clonal population. During its potentially life-long association with the human host, *C. albicans* frequently faces environmental changes to which it must adapt. This may result in the emergence of genetically altered variants that are better able to cope with new adverse conditions. Since there is often more than one way to deal with a particular stress, individual cells in the population may come up with different solutions, and a combination of their adaptive traits could further increase the fitness of the cells. An illustrative example is the development of fluconazole resistance during antifungal therapy of patients suffering from candidiasis (23). Fluconazole inhibits the biosynthesis of ergosterol, the main sterol in fungal membranes, by targeting the enzyme sterol 14α-demethylase. *C. albicans* can acquire fluconazole resistance by various mechanisms. Mutations in the *ERG11* gene encoding sterol 14α-demethylase, which result in reduced drug binding, increase the resistance of the cells to fluconazole (24–28). Overexpression of *ERG11* also confers increased fluconazole resistance and is frequently caused by gain-of-function (GOF) mutations in the transcription factor Upc2 (29–33). Similarly, GOF mutations in the transcription factors Mrr1 and Tac1 result in overexpression of their target genes, including the multidrug efflux pumps *MDR1* and *CDR1*/*CDR2*, respectively, and cause fluconazole resistance (9, 10, 33–40). Combinations of these resistance mechanisms potentiate drug resistance and are commonly found in highly fluconazole-resistant clinical isolates (24, 25, 27, 33, 36, 41–43). Such isolates are usually homozygous for the resistance mutations, because loss of heterozygosity (LOH) for a mutated allele further enhances drug resistance (42), and fluconazole and other stresses increase the frequency of such genomic alterations (44, 45). As LOH involves mitotic recombination or whole-chromosome loss, many additional genomic loci are usually affected. *TAC1* is closely linked to *MTL* on the left arm of chromosome 5, and LOH for a mutated *TAC1* allele is often associated with *MTL* homozygosity (6, 9, 10, 34). Furthermore, whole-chromosome loss by missegregation may involve more than one chromosome (8, 12), and LOH events at one locus are frequently accompanied by LOH at additional loci and chromosomes (46), which increases the chances that other chromosomes or parts thereof also become homozygous when LOH for an acquired resistance mutation is favored under drug selection. These observations raise the intriguing hypothesis that, in an originally clonal population, those cells that have acquired a resistance mutation and subsequently become homozygous for the mutated allele are likely to have become mating-competent and can therefore combine their individually gained resistance mechanisms by parasexual recombination to produce highly drug-resistant cells.

In our present study, we have addressed this hypothesis using a set of defined strains containing different fluconazole resistance mutations. We show that in the presence of the drug, heterozygous strains quickly generate derivatives that are homozygous for the mutated allele and for *MTL*. These cells can mate with each other and then reassort the combined chromosome sets to produce highly drug-resistant progeny. Our results demonstrate that parasexual recombination, promoted by stress-induced genomic rearrangements that result in the acquisition of mating competence in cells with adaptive mutations, may be an important mechanism in the evolution of *C. albicans* populations.

## RESULTS

### Fluconazole promotes loss of heterozygosity in *C. albicans* strains with different drug resistance mechanisms.

**FIG 1:**
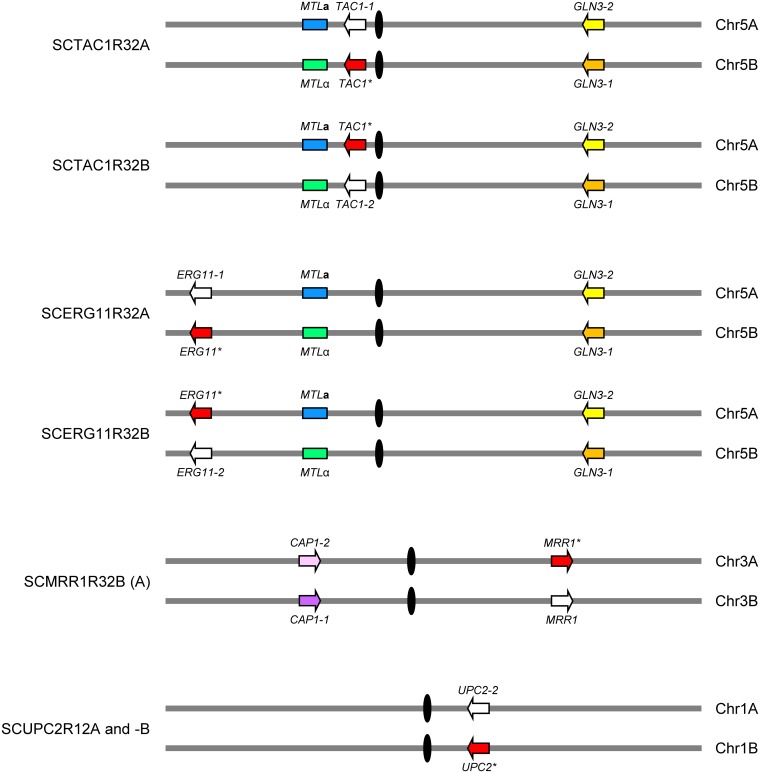
Schematic showing the location of mutated *TAC1*, *ERG11*, *MRR1*, and *UPC2* alleles on the respective chromosomes in the genetically engineered *C. albicans* strains used in this study. Genes (arrows) and chromosomes (gray lines) are not drawn to scale; only the relative positions of the genes, *MTL***a** and *MTL*α loci (blue and green rectangles, respectively), and centromeres (black ovals) are indicated. Alleles carrying fluconazole resistance mutations (marked by stars) are represented by red arrows, and the corresponding wild-type alleles on the homologous chromosomes by white arrows. The polymorphic *GLN3* and *CAP1* alleles on the right arm of Chr5 and on the left arm of Chr3, respectively, which were used to analyze LOH events, are also shown and distinguished by color. Note that the parental strain SC5314 is almost completely homozygous for the right arm of Chr3; the putative location of the mutated *MRR1* allele on Chr3A in strain SCMRR1R32B was deduced from its linkage to the *CAP1-2* allele after an LOH event in homozygous derivatives; for strain SCMRR1R32A no such derivative was obtained, and the location of the mutated *MRR1* on Chr3A or Chr3B in this strain has not been established.

In order to screen for fluconazole-induced LOH events, we used a set of eight isogenic strains, all derived from the fluconazole-susceptible *C. albicans* reference strain SC5314, which were heterozygous for resistance mutations in *TAC1*, *ERG11*, *MRR1*, or *UPC2* (Fig. 1 and Table 1). Strain SCTAC1R32A contains a G980E GOF mutation in the *TAC1* allele that is linked to *MTL*α on the left arm of Chr5B, whereas strain SCTAC1R32B contains the same mutation in the *TAC1* allele that is linked to *MTL***a** on the homologous Chr5A. Similarly, strain SCERG11R32A contains a G464S mutation in the *ERG11* allele that is linked to *MTL*α on the left arm of Chr5B, while strain SCERG11R32B contains the same mutation in the *ERG11* allele that is linked to *MTL***a** on Chr5A. *MRR1* is not linked to *MTL* and located on the right arm of Chr3, which is almost completely homozygous in strain SC5314, so that the two alleles cannot be distinguished. Strains SCMRR1R32A and -B are two independently constructed mutants containing a P683S GOF mutation in one of the *MRR1* alleles. *UPC2* also is not linked to *MTL* and located on the right arm of Chr1; the two independently generated strains SCUPC2R12A and -B contain a G648D GOF mutation in the *UPC2* allele on Chr1B.

**TABLE 1:** Genetically engineered *C. albicans* strains used in this study

Strain	Parent	Relevant genotype^*a*^	Fluconazole MIC (μg/ml)	Reference
SC5314		Wild-type reference strain	0.5	65
SCERG11R32A	SC5314	*ERG11-1*/*ERG11*^G464S^	2	42
SCERG11R32B	SC5314	*ERG11*^G464S^/*ERG11-2*	2l	42
SCERG11R34A	SCERG11R32A	*ERG11*^G464S^/*ERG11*^G464S^	4	42
SCERG11R34B	SCERG11R32B	*ERG11*^G464S^/*ERG11*^G464S^	4	42
SCMRR1R32A	SC5314	*MRR1*/*MRR1*^P683S^	4	62
SCMRR1R32B	SC5314	*MRR1*/*MRR1*^P683S^	4	62
SCMRR1R34A	SCMRR1R32A	*MRR1*^P683S^/*MRR1*^P683S^	16	62
SCMRR1R34B	SCMRR1R32B	*MRR1*^P683S^/*MRR1*^P683S^	16	62
SCTAC1R32A	SC5314	*TAC1-1*/*TAC1*^G980E^	2	60
SCTAC1R32B	SC5314	*TAC1*^G980E^/*TAC1-2*	2	60
SCTAC1R34A	SCTAC1R32A	*TAC1*^G980E^/*TAC1*^G980E^	8	60
SCTAC1R34B	SCTAC1R32B	*TAC1*^G980E^/*TAC1*^G980E^	8	60
SCUPC2R12A	SC5314	*UPC2*^G648D^/*UPC2*-*2*	2	31
SCUPC2R12B	SC5314	*UPC2*^G648D^/*UPC2*-*2*	2	31
SCUPC2R14A	SCUPC2R12A	*UPC2*^G648D^/*UPC2*^G648D^	4	31
SCUPC2R14B	SCUPC2R12B	*UPC2*^G648D^/*UPC2*^G648D^	4	31
SCETR34A	SCERG11R34A	*ERG11*^G464S^/*ERG11*^G464S^; *TAC1*^G980E^/*TAC1*^G980E^	16	42
SCETR34B	SCERG11R34B	*ERG11*^G464S^/*ERG11*^G464S^; *TAC1*^G980E^/*TAC1*^G980E^	16	42
SCEUR14A	SCERG11R34A	*ERG11*^G464S^/*ERG11*^G464S^; *UPC2*^G648D^/*UPC2*^G648D^	16	42
SCEUR14A	SCERG11R34B	*ERG11*^G464S^/*ERG11*^G464S^; *UPC2*^G648D^/*UPC2*^G648D^	16	42
SCMER34A	SCMRR1R34A	*MRR1*^P683S^/*MRR1*^P683S^; *ERG11*^G464S^/*ERG11*^G464S^	64	42
SCMER34B	SCMRR1R34B	*MRR1*^P683S^/*MRR1*^P683S^; *ERG11*^G464S^/*ERG11*^G464S^	64	42
SCMTR34A	SCMRR1R34A	*MRR1*^P683S^/*MRR1*^P683S^; *TAC1*^G980E^/*TAC1*^G980E^	32	42
SCMTR34B	SCMRR1R34B	*MRR1*^P683S^/*MRR1*^P683S^; *TAC1*^G980E^/*TAC1*^G980E^	32	42
SCMUR14A	SCMRR1R34A	*MRR1*^P683S^/*MRR1*^P683S^; *UPC2*^G648D^/*UPC2*^G648D^	64	42
SCMUR14B	SCMRR1R34B	*MRR1*^P683S^/*MRR1*^P683S^; *UPC2*^G648D^/*UPC2*^G648D^	64	42
SCUTR34A	SCUPC2R14A	*UPC2*^G648D^/*UPC2*^G648D^; *TAC1*^G980E^/*TAC1*^G980E^	64	42
SCUTR34B	SCUPC2R14B	*UPC2*^G648D^/*UPC2*^G648D^; *TAC1*^G980E^/*TAC1*^G980E^	64	42
SCEThet1A	SCERG11R32A	*ERG11-1*/*ERG11*^G464S^; *TAC1*^G980E^/*TAC1-2*	4	This study
SCEThet1B	SCERG11R32B	*ERG11*^G464S^/*ERG11-2*; *TAC1*^G980E^/*TAC1-2*	4	This study
SCMEhet1A	SCMRR1R32A	*MRR1*/*MRR1*^P683S^; *ERG11*^G464S^/*ERG11-2*	8	This study
SCMEhet1B	SCMRR1R32B	*MRR1*/*MRR1*^P683S^; *ERG11-1*/*ERG11*^G464S^	8	This study
SCMThet1A	SCMRR1R32A	*MRR1*/*MRR1*^P683S^; *TAC1*^G980E^/*TAC1-2*	8	This study
SCMThet1B	SCMRR1R32B	*MRR1*/*MRR1*^P683S^; *TAC1-1*/*TAC1*^G980E^	8	This study
SCMUhet1A	SCMRR1R32A	*MRR1*/*MRR1*^P683S^; *UPC2*^G648D^/*UPC2*-*2*	8	This study
SCMUhet1B	SCMRR1R32B	*MRR1*/*MRR1*^P683S^; *UPC2*^G648D^/*UPC2*-*2*	8	This study
SCUEhet1A	SCUPC2R12A	*UPC2*^G648D^/*UPC2*-*2*; *ERG11-1*/*ERG11*^G464S^	4	This study
SCUEhet1B	SCUPC2R12B	*UPC2*^G648D^/*UPC2*-*2*; *ERG11-1*/*ERG11*^G464S^	4	This study
SCUThet1A	SCUPC2R12A	*UPC2*^G648D^/*UPC2*-*2*; *TAC1-1*/*TAC1*^G980E^	8	This study
SCUThet1B	SCUPC2R12B	*UPC2*^G648D^/*UPC2*-*2*; *TAC1-1*/*TAC1*^G980E^	8	This study
SCMTLαM2A	SC5314	*MTL***a**/*mtl*αΔ	0.5	66
SCMTL**a**M2A	SC5314	*mtl***a**Δ/*MTL*α	0.5	66

a The two alleles of *ERG11*, *TAC1*, and *UPC2* in strain SC5314 were distinguished by HindIII, SpeI, and EcoRI restriction site polymorphisms, respectively. The *ERG11* allele with the HindIII site at position +347 (on Chr5B) was arbitrarily designated *ERG11-2* (42), the *TAC1* allele with the downstream SpeI site at position +4411 (on Chr5B) was designated *TAC1-2* (60), and the *UPC2* allele with the EcoRI site at position +1593 (on Chr1A) was designated *UPC2-2* (31).

We first tested whether derivatives that had become homozygous for the different fluconazole resistance mutations would be distinguishable from their heterozygous progenitors by improved growth (i.e., colony size) on fluconazole-containing agar plates. To this aim, we compared the growth of genetically engineered strains into which the mutations had been introduced into one or both of the respective alleles (Table 1) on plates with and without fluconazole. As can be seen in Fig. S1 in the supplemental material, strains containing the *TAC1* or *MRR1* GOF mutations in both alleles grew much better than their heterozygous counterparts in the presence of the drug, indicating that screening for larger colonies on such plates should allow the identification of homozygous derivatives. In contrast, strains that were homozygous for the *ERG11* or *UPC2* mutations could not be distinguished from heterozygous strains under these conditions, despite the slightly elevated MICs of the homozygous strains in broth microdilution assays, so that a different screening procedure was necessary in these cases.

10.1128/mBio.02740-18.1 FIG S1 Growth of strains with heterozygous and homozygous resistance mutations on agar plates containing fluconazole. Download FIG S1, PDF file, 0.2 MB. Copyright © 2019 Popp et al. 2019 Popp et al. This content is distributed under the terms of the Creative Commons Attribution 4.0 International license.

**FIG 2:**
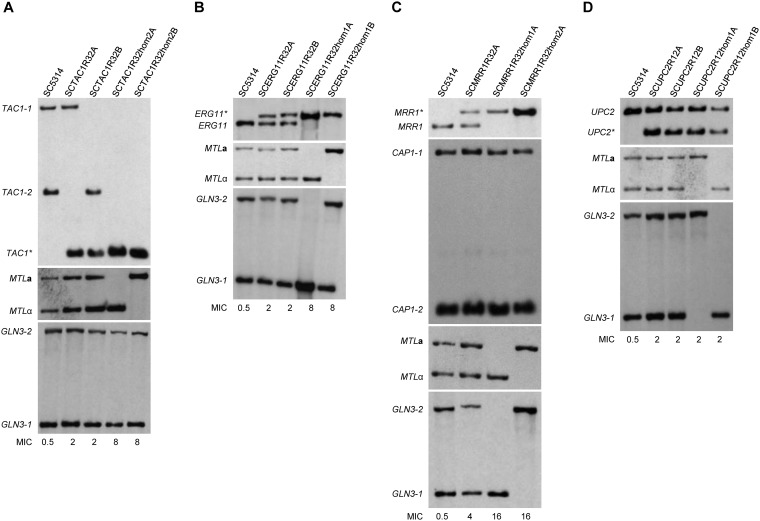
Genetic analysis of fluconazole-induced *MTL*-homozygous strains. Shown are the genetically engineered strains that are heterozygous for mutated TAC1* (SCTAC1R32A and -B), ERG11* (SCERG11R32A and -B), MRR1* (SCMRR1R32A), and UPC2* (SCUPC2R12A and -B) alleles and derivatives that became homozygous for *MTL***a** or *MTL*α after growth in the presence of fluconazole. The parental wild-type reference strain SC5314 is included for comparison. A schematic showing the restriction site polymorphisms used to distinguish wild-type and mutated *ERG11*, *MRR1*, *TAC1*, and *UPC2* alleles, the polymorphic wild-type *GLN3* and *CAP1* alleles, and the *MTL***a** and *MTL*α loci, as well as the expected fragment sizes after hybridization with specific probes is presented in Fig. S2 in the supplemental material. Some blots are from independent genomic DNA preparations of the same strains, and differences in signal intensities are caused by unequal loading. The MIC (μg/ml) of fluconazole for each strain is given below the corresponding lane.

**(i) *MTL*-homozygous strains with a resistance mutation in *TAC1*.** Strains SCTAC1R32A and -B, which are heterozygous for the *TAC1* GOF mutation, were serially passaged in YPD medium containing 5 µg/ml fluconazole, and appropriate dilutions of each subculture were plated on YPD agar with 5 µg/ml fluconazole. After only two passages in the presence of the drug, the populations contained cells that produced larger colonies on the fluconazole plates, indicating that these cells had acquired enhanced drug resistance. We picked twelve large colonies (six from strain A and six from strain B) for genetic analysis. A preliminary PCR analysis showed that seven of the twelve clones had lost the wild-type *TAC1* allele and contained only the mutated *TAC1* allele, suggesting that LOH for the GOF mutation was the predominant mechanism of increased fluconazole resistance in these strains. As expected from the close linkage of the *TAC1* and *MTL* loci, the three clones derived from strain A contained only *MTL*α but not *MTL***a**, and the four clones derived from strain B contained only *MTL***a** but not *MTL*α. For all seven clones, the MIC of fluconazole had increased from 2 µg/ml to 8 µg/ml, as for strains SCTAC1R34A and -B in which the same resistance mutation had been sequentially introduced into both *TAC1* alleles by genetic engineering. To investigate whether the LOH for *MTL* and the mutated *TAC1* allele had occurred by mitotic recombination or chromosome loss, we tested the seven clones for the presence of a restriction site polymorphism upstream of *GLN3* on the right arm of Chr5 (Fig. 1). All three clones derived from strain A and one of the four clones derived from strain B had retained the polymorphism, excluding whole-chromosome loss as the mechanism of LOH. The remaining three clones contained only the *GLN3-2* allele, which like *MTL***a** is located on Chr5A, indicating that the LOH may have been caused by a whole-chromosome loss in these strains, although multiple recombination events involving both chromosome arms cannot be excluded by this simple test. The results of the genetic and phenotypic analysis of the seven homozygous clones are summarized in Table S1A. We selected one *MTL*α and one *MTL***a** strain, both of which had retained heterozygosity on the other Chr5 arm, for our further experiments (Table 2). A Southern hybridization analysis demonstrating the genomic alterations in strains SCTAC1R32hom2A and SCTAC1R32hom2B is presented in Fig. 2A.

**TABLE 2:** Fluconazole-induced *MTL*-homozygous strains used in mating experiments

Strain	Parent	Relevant genotype^*a*^	Fluconazole MIC (µg/ml)
SCERG11R32hom1A	SCERG11R32A	*ERG11*^G464S^/*ERG11*^G464S^; *MTL*α/α; *GLN3-1*/*GLN3-1*	8
SCERG11R32hom1B	SCERG11R32B	*ERG11*^G464S^/*ERG11*^G464S^; *MTL***a**/**a**; *GLN3-1*/*GLN3-2*	8
SCMRR1R32hom1A	SCMRR1R32A	*MRR1*^P683S^/*MRR1*^P683S^; *MTL*α/α; *GLN3-1*/*GLN3-1*; *CAP1-1*/*CAP1-2*	16
SCMRR1R32hom2A	SCMRR1R32A	*MRR1*^P683S^/*MRR1*^P683S^; *MTL***a**/**a**; *GLN3-2*/*GLN3-2*; *CAP1-1*/*CAP1-2*	16
SCTAC1R32hom2A	SCTAC1R32A	*TAC1*^G980E^/*TAC1*^G980E^; *MTL*α/α; *GLN3-1*/*GLN3-2*	8
SCTAC1R32hom2B	SCTAC1R32B	*TAC1*^G980E^/*TAC1*^G980E^; *MTL***a**/**a**; *GLN3-1*/*GLN3-2*	8
SCUPC2R12hom1A	SCUPC2R12A	*UPC2*^G648D^/*UPC2*-*2*; *MTL***a**/**a**; *GLN3-2*/*GLN3-2*	2
SCUPC2R12hom1B	SCUPC2R12B	*UPC2*^G648D^/*UPC2*-*2*; *MTL*α/α; *GLN3-1*/*GLN3-1*	2

a The two *GLN3* alleles on the right arm of Chr5 in strain SC5314 were distinguished by a ClaI restriction site polymorphism; the *GLN3* allele with the upstream ClaI site at position −1712 (on Chr5B) was designated *GLN3-1* (67). The two *CAP1* alleles on the left arm of Chr3 in strain SC5314 were distinguished by an EcoRI restriction site polymorphism; the *CAP1* allele with the EcoRI site at position +1212 (on Chr3A) was designated *CAP1-2* (62).

10.1128/mBio.02740-18.2 FIG S2 Discrimination between wild-type and mutated *ERG11*, *MRR1*, *TAC1*, and *UPC2* alleles and between the polymorphic wild-type *GLN3*, *CAP1*, and *MTL* alleles in derivatives of strain SC5314 by Southern hybridization. Download FIG S2, PDF file, 0.1 MB. Copyright © 2019 Popp et al. 2019 Popp et al. This content is distributed under the terms of the Creative Commons Attribution 4.0 International license.

**(ii) *MTL*-homozygous strains with a resistance mutation in *ERG11*.** As explained above, strains that are heterozygous or homozygous for the *ERG11*^G464S^ allele could not be distinguished on fluconazole-containing agar plates (see Fig. S1). Since *ERG11* is located on the same chromosome arm as *MTL* (Fig. 1), LOH for the mutated *ERG11* allele would be expected to be frequently accompanied by *MTL* homozygosity. We therefore screened for *MTL*-homozygous derivatives that emerged after drug exposure. *MTL*-homozygous cells switch from the white to the opaque state when incubated on solid media at high CO_2_ concentrations (47), allowing their identification by the characteristic opaque colony phenotype. Strains SCERG11R32A and -B, which are heterozygous for the *ERG11*^G464S^ allele, were serially passaged in YPD medium containing 2.5 µg/ml fluconazole. Appropriate dilutions of each subculture were plated on Lee’s agar containing phloxine B, which stains opaque colonies pink, and incubated for 2 days at 25°C in a 18% CO_2_ atmosphere to induce switching of *MTL*-homozygous cells to the opaque phase. After an additional incubation for 5 days at room temperature in a normal atmosphere, opaque colonies were picked and characterized. All 14 analyzed opaque colonies from different passages of strain SCERG11R32A had lost *MTL***a** and the wild-type *ERG11* allele and contained only *MTL*α and the linked *ERG11*^G464S^ allele (Table S1A). The clones also retained only the *GLN3-1* allele on the other arm of Chr5B, indicating that loss of the whole Chr5A or multiple recombination events had occurred in these cells (we note that some or even all of these clones could be descendants of the same progenitor, because they came from the same starting culture). Of 20 analyzed *MTL*-homozygous clones that were obtained from different passages of strain SCERG11R32B, 15 were also homozygous for the mutated *ERG11* allele. The other five clones were homozygous for *MTL*α but still contained both the wild-type and the mutated *ERG11* alleles, demonstrating that in these cases LOH for *MTL* occurred by a recombination event that did not involve the *ERG11* locus on the same chromosome arm. All 15 clones that were homozygous for both *MTL* and the mutated *ERG11* allele retained the polymorphism near *GLN3* on the other chromosome arm, excluding chromosome loss as the mechanism of LOH. Interestingly, only eleven of these clones retained *MTL***a**, which was linked to the *ERG11* mutation in the parental strain, whereas the other four clones were homozygous for *MTL*α and the mutated *ERG11* allele, pointing to more complex recombination events. For all clones that had become homozygous for the *ERG11*^G464S^ allele, the MIC of fluconazole increased from 2 µg/ml to 8 µg/ml, slightly higher than for strains SCERG11R34A and -B in which the G464S mutation had been sequentially introduced into both *ERG11* alleles by genetic engineering. We selected one *MTL*α strain (SCERG11R32hom1A) and one *MTL***a** strain (SCERG11R32hom1B) for our further experiments (Table 2). A Southern hybridization analysis documenting the genomic alterations in these strains is presented in Fig. 2B.

**(iii) *MTL*-homozygous strains with a resistance mutation in *MRR1*.** Although strains that are homozygous for the *MRR1*^P683S^ mutation could be easily distinguished from heterozygous strains on fluconazole-containing agar plates (Fig. S1), LOH for the mutated *MRR1* allele would be expected to be accompanied by *MTL* homozygosity only in a minority of such events, because the two loci are on different chromosomes. We therefore decided to directly screen for switching-competent cells after growth of the heterozygous strains SCMRR1R32A and -B in the presence of fluconazole. The strains were serially passaged in YPD medium containing 1.75 µg/ml or 2.5 µg/ml fluconazole, and screening for opaque colonies was performed as described above for the strains containing a resistance mutation in *ERG11*. We recovered three *MTL*-homozygous derivatives from strain A (two *MTL*α, one *MTL***a**) and three from strain B (one *MTL*α, two *MTL***a**) (see Table S1A). All six *MTL*-homozygous clones were also homozygous for the *MRR1* GOF mutation, indicating that LOH for the mutated *MRR1* allele was the driving force that enriched *MTL*-homozygous cells in the population. Four of the six clones retained both polymorphic *CAP1* alleles on the other Chr3 arm (Fig. 1), demonstrating that LOH for the *MRR1* mutation was caused by mitotic recombination instead of whole-chromosome loss. The other two clones, both derived from strain B, retained only the *CAP1-2* allele and may have resulted from a loss of Chr3B. In contrast, *MTL* homozygosity was in all six clones accompanied by LOH for the *GLN3* polymorphism on the other Chr5 arm and showed the expected linkage of *MTL*α with the *GLN3-1* allele and of *MTL***a** with *GLN3-2*. *MTL* homozygosity therefore may have been caused in all cases by loss of the other Chr5 homolog. The MIC of fluconazole had increased from 4 µg/ml to 16 µg/ml for all six clones, as for strains SCMRR1R34A and -B in which the P683S mutation had been sequentially introduced into both *MRR1* alleles by genetic engineering. We selected one *MTL*α strain (SCMRR1R32hom1A) and one *MTL***a** strain (SCMRR1R32hom2A) for further experiments (Table 2); Fig. 2C shows the relevant genomic alterations in these strains.

**(iv) *MTL*-homozygous strains with a resistance mutation in *UPC2*.** As *UPC2* is located on Chr1 and not linked to *MTL*, we also screened for switching-competent cells after passaging strains SCUPC2R12A and -B, which are heterozygous for the *UPC2*^G648D^ allele, in YPD medium containing 2.5 µg/ml or 5 µg/ml fluconazole. Eight *MTL*-homozygous clones (six *MTL***a**, two *MTL*α) could be isolated from different passages of strain A, and one *MTL*-homozygous derivative (*MTL*α) was obtained from strain B. None of these clones had become homozygous for the mutated *UPC2* allele, indicating that the minor increase in fluconazole resistance of strains containing the GOF mutation in both *UPC2* alleles instead of only one was not enough to efficiently select for this event. Eight of the nine *MTL*-homozygous strains were also homozygous for the corresponding *GLN3* allele on the other Chr5 arm, indicating that loss of the homologous chromosome, induced by fluconazole stress, may have caused LOH in the majority of cases. Except for one clone, in which the MIC of fluconazole increased by an unknown mutation, all *MTL*-homozygous clones displayed the same fluconazole susceptibility as the heterozygous parental strains (see Table S1A). Although a more extensive screening might have resulted in the isolation of derivatives that are also homozygous for the mutated *UPC2* alleles, we decided to use heterozygous strains for mating experiments and selected one *MTL***a** strain (SCUPC2R12hom1A) and one *MTL*α strain (SCUPC2R12hom1B) for this purpose (Table 2 and Fig. 2D).

10.1128/mBio.02740-18.9 TABLE S1 (A) Fluconazole-induced *MTL*-homozygous *C. albicans* strains. (B) Mating products obtained from different crosses. (C) Fluconazole susceptibilities of mating products that are homozygous or heterozygous for single resistance mutations. (D) Mating product progeny exhibiting increased drug resistance after passage in the presence of fluconazole. Download Table S1, XLSX file, 0.03 MB. Copyright © 2019 Popp et al. 2019 Popp et al. This content is distributed under the terms of the Creative Commons Attribution 4.0 International license.

### *MTL*-homozygous *C. albicans* strains can combine their resistance mutations by mating.

To investigate if *C. albicans* strains exhibiting several fluconazole resistance mechanisms can be generated by parasexual recombination, we performed mating experiments between strains with different resistance mutations and opposite *MTL* configurations. For example, the *MTL***a** strain with the *ERG11*^G464S^ mutation was mated with the three *MTL*α strains containing the *MRR1*^P683S^, *TAC1*^G980E^, and *UPC2*^G648D^ mutations, and the *MTL*α strain with the *ERG11*^G464S^ mutation was mated with the corresponding three *MTL***a** partners. Altogether, twelve different matings were performed to test all possible combinations of our selected strains.

**FIG 3:**
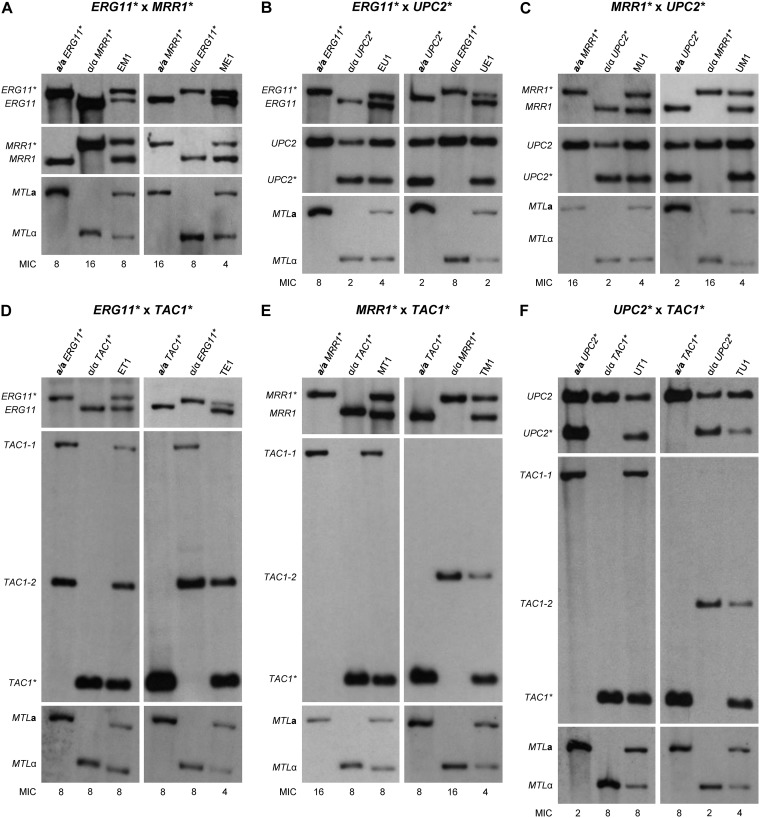
Genetic analysis of mating products by Southern hybridization with relevant probes. All mating products and their parental strains are listed in Table 3. The *MTL* configuration (**a**/**a** or α/α) and fluconazole resistance alleles (*) of the parental strains are indicated. See Fig. S2 for a schematic of the loci. The names of the mating products indicate the resistance alleles (E for ERG11*, M for MRR1*, T for TAC1*, U for UPC2*) and which of them came from the **a**/**a** parent (first letter) and which from the α/α parent (second letter). Some blots are from independent genomic DNA preparations of the same strains, and differences in signal intensities are caused by unequal loading. The MIC (μg/ml) of fluconazole for each strain is given below the corresponding lane.

Mating experiments with *C. albicans* are usually performed by using partner strains with different auxotrophies to enable the selection of prototrophic mating products on minimal plates lacking the necessary supplements for growth of the parental strains. As our strains were all prototrophic and also did not contain dominant selection markers, we used a different strategy to identify mating products. Opaque cells of the mating partners were mixed 1:1, spotted on Spider agar plates, and first incubated for 2 days at 25°C in an 18% CO_2_ atmosphere, followed by further incubation at room temperature in normal air. Samples of the cell lawns were taken on different days, spread for single colonies on Lee’s agar plates with phloxine B, and incubated for 2 days at 25°C in an 18% CO_2_ atmosphere and then for another 5 days at room temperature in normal air. Under these conditions, the parental strains should stay in the opaque phase whereas *MTL*-heterozygous mating products would switch back to the white phase and be identifiable as white colonies. Therefore, white colonies were picked and first analyzed for the presence of both *MTL* alleles. Despite some background of parental cells forming white colonies, the frequency of which varied from strain to strain, we could successfully isolate mating products from all crosses (Table S1B). Southern hybridization analysis showed that all tested mating products contained the genetic material from both parents, i.e., *MTL***a** and *MTL*α loci as well as the two different fluconazole resistance genes and the corresponding wild-type alleles (Fig. 3 shows an example of each cross).

Despite the fact that the mating products contained the fluconazole resistance genes from both parents, they did not display increased drug resistance and usually were even more susceptible than one or both of the parental strains. This can be explained by the fact that the mating products also contained the corresponding wild-type alleles; it is conceivable that heterozygosity for resistance genes reduces the phenotypic effect of the resistance mutations not only in diploid strains (Table 1) but also in tetraploid mating products and also when strains are heterozygous instead of homozygous for two different resistance mutations. We tested this assumption in two ways. First, we constructed a set of double-heterozygous diploid strains containing all possible combinations of two different resistance mutations. As summarized in Table 1, the double-heterozygous strains were less resistant to fluconazole than the corresponding double-homozygous strains, and in some cases even less resistant than strains that were homozygous for only one of the two mutations. In a second experiment, we mated the fluconazole-selected *MTL*-homozygous strains containing GOF mutations in *MRR1* or *TAC1* with strains of the opposite mating type containing the same resistance mutation or the corresponding wild-type alleles. Mating products were identified and genetically analyzed as described above, and their fluconazole susceptibilities were determined (Table S1C). As expected, tetraploid strains containing two mutated and two wild-type *MRR1* or *TAC1* alleles were more resistant to fluconazole than tetraploid control strains containing only wild-type alleles but less resistant than tetraploid strains in which all four alleles of the respective gene contained the resistance mutation.

**FIG 4:**
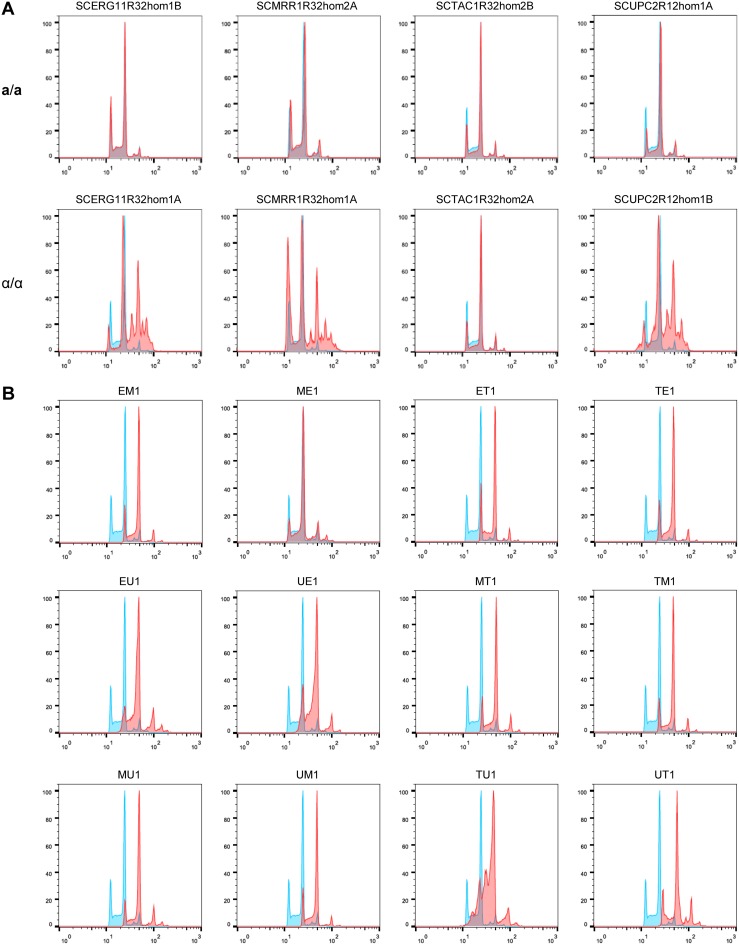
Ploidy analysis of mating products and their parental strains. The plots show the results of flow cytometric measurements of the DNA content of the fluconazole-induced *MTL*-homozygous strains (A) and the mating products obtained in the various crossings (B). The profile of the diploid reference strain SC5314 is shown in blue, and the profiles of all other strains are shown in red.

As explained in the introduction, tetraploid cells are not stable and can lose chromosomes to become aneuploid and eventually revert to the diploid state. We reasoned that upon further propagation in the presence of fluconazole, the mating products would evolve increased drug resistance by reassorting their combined chromosome sets in an advantageous way. Therefore, we selected one mating product from each cross (those listed in Table 3 and shown in Fig. 3) to test this hypothesis (see below). We first analyzed the ploidy of the selected original mating products and their parental strains by flow cytometry. The starting strains that were heterozygous for the various resistance mutations exhibited the same 2C and 4C peaks as the reference strain SC5314, from which they were derived and therefore were diploid as expected (Fig. S3). In contrast, an altered ploidy was observed for some of the fluconazole-induced *MTL*-homozygous strains (Fig. 4A). While SCERG11R32hom1B, SCTAC1R32hom2A, and SCTAC1R32hom2B appeared diploid, SCMRR1R32hom2A and SCUPC2R12hom1A had a slightly higher than 4N DNA content, and SCERG11R32hom1A, SCMRR1R32hom1A, and SCUPC2R12hom1B apparently contained a subpopulation of cells with various ploidies. Most of the mating products displayed a shift of the peaks that was compatible with the expected tetraploidy (Fig. 4B). However, mating product ME1 appeared to be diploid, whereas mating product UT1 was hypertetraploid, and TU1 showed population heterogeneity, indicating that the mating products were not stable and had undergone genomic changes during the following cell divisions before they were isolated and analyzed. Variability in the ploidy of some mating products was even seen when independent subcultures of the same frozen stocks were analyzed by flow cytometry (Fig. S4).

**TABLE 3:** Mating products used for passaging experiments

Mating product	*MTL*a/a parent	*MTL*α/α parent	Fluconazole MIC (µg/ml)
EM1	SCERG11R32hom1B	SCMRR1R32hom1A	8
ME1	SCMRR1R32hom2A	SCERG11R32hom1A	4
ET1	SCERG11R32hom1B	SCTAC1R32hom2A	8
TE1	SCTAC1R32hom2B	SCERG11R32hom1A	4
EU1	SCERG11R32hom1B	SCUPC2R12hom1B	4
UE1	SCUPC2R12hom1A	SCERG11R32hom1A	2
MT1	SCMRR1R32hom2A	SCTAC1R32hom2A	8
TM1	SCTAC1R32hom2B	SCMRR1R32hom1A	4
MU1	SCMRR1R32hom2A	SCUPC2R12hom1B	4
UM1	SCUPC2R12hom1A	SCMRR1R32hom1A	4
TU1	SCTAC1R32hom2B	SCUPC2R12hom1B	4
UT1	SCUPC2R12hom1A	SCTAC1R32hom2A	8

10.1128/mBio.02740-18.3 FIG S3 Ploidy analysis of *C. albicans* strains containing different fluconazole resistance mutations. Download FIG S3, PDF file, 0.1 MB. Copyright © 2019 Popp et al. 2019 Popp et al. This content is distributed under the terms of the Creative Commons Attribution 4.0 International license.

10.1128/mBio.02740-18.4 FIG S4 Ploidy analysis of mating products. Download FIG S4, PDF file, 0.3 MB. Copyright © 2019 Popp et al. 2019 Popp et al. This content is distributed under the terms of the Creative Commons Attribution 4.0 International license.

**FIG 5:**
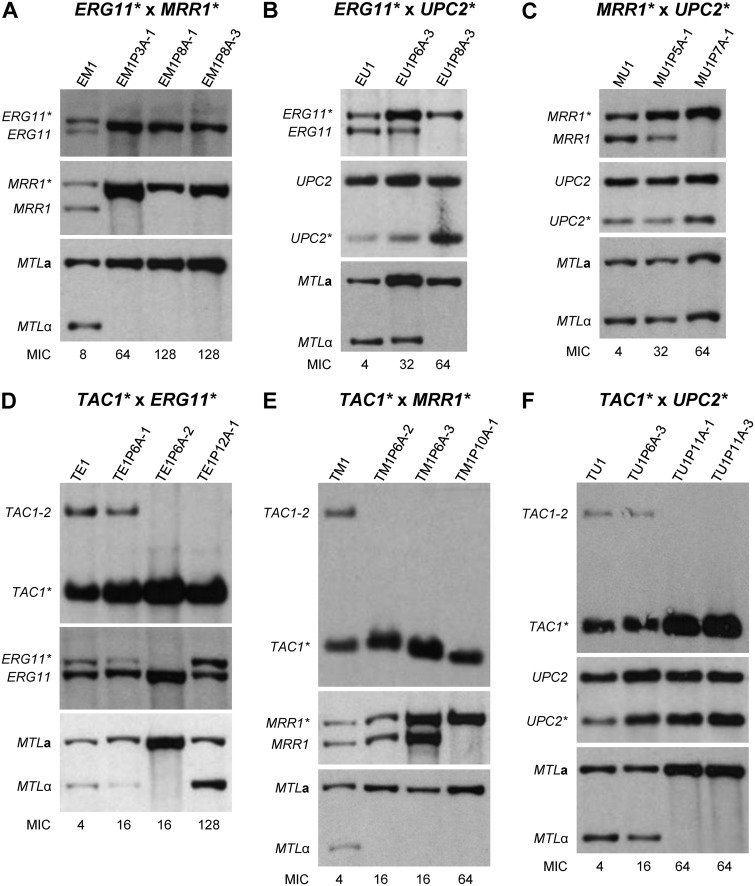
Genetic analysis of mating product progeny by Southern hybridization with relevant probes. Shown are original mating products (first lanes) and derivatives exhibiting increased fluconazole resistance after passage in the presence of the drug. Progeny of mating products of the reverse crossings are presented in Fig. S5. Some blots are from independent genomic DNA preparations of the same strains, and differences in signal intensities are caused by unequal loading. The MIC (μg/ml) of fluconazole for each strain is given below the corresponding lane. The observed genetic changes are summarized in Table 4.

A partial loss of specific alleles in some mating products was also evident from the Southern hybridization analyses (Fig. 3 and 5 and Fig. S5). The weaker signal of the mutated ERG11* allele compared with the wild-type *ERG11* allele in mating products TE1 (Fig. 3D) and UE1 (Fig. 3B) indicated a reduced copy number of ERG11*, and this coincided with a weaker signal of the linked *MTL*α allele compared to *MTL***a** in these strains. Mating product TE1 also contained only one of the two wild-type *TAC1* alleles from its α/α parent. Conversely, mating product EM1 contained more mutated ERG11* than wild-type *ERG11*, and the signal of the linked *MTL***a** was also stronger than that of *MTL*α (Fig. 3A). Therefore, these mating products may already have lost one copy of Chr5B at the time when they were isolated and analyzed, although chromosome loss was not evident from the flow cytometry analysis (Fig. 4B and Fig. S4). Mating product ME1 exhibited a weaker signal for the mutated MRR1* allele than for wild-type *MRR1* (Fig. 3A), in contrast to the other mating products containing MRR1*, pointing to a possible loss of one copy of Chr3 (note that ME1 appeared diploid in the flow cytometry analysis [Fig. 4B]). Another noticeable example is mating product UT1, which appears to contain similar amounts of wild-type *UPC2* and the mutated UPC2* allele (Fig. 3F), although a 3:1 ratio would be expected in a tetraploid mating product in this case, because the parental strain containing UPC2* was heterozygous (compare with the other mating products containing UPC2* in Fig. 3 and 5 and Fig. S5; the differences are better seen in the latter figures). Therefore, UT1 may have lost two copies of Chr1 with wild-type *UPC2*, undergone a recombination event that replaced one wild-type *UPC2* copy with UPC2*, or amplified the chromosome containing UPC2* (note that UT1 appeared to have slightly higher than 4N genome content [Fig. 4B]). The finding that the mating products isolated in our present study seem to be less stable than those that were analyzed in previous reports by other researchers is most likely due to the fact that no selection pressure for maintenance of specific markers was applied in our experiments and the mating products could immediately lose chromosomes in a random fashion in subsequent cell divisions.

10.1128/mBio.02740-18.5 FIG S5 Genetic analysis of mating product progeny by Southern hybridization with relevant probes. Download FIG S5, PDF file, 0.2 MB. Copyright © 2019 Popp et al. 2019 Popp et al. This content is distributed under the terms of the Creative Commons Attribution 4.0 International license.

### Mating product progeny acquire high levels of drug resistance by selective chromosome loss.

In order to test whether the mating products could reassort their combined chromosome sets in such a way that they acquired enhanced fluconazole resistance under selective pressure, we serially passaged them in media containing increasing fluconazole concentrations, starting with a concentration that was half the MIC and doubling it on every second passage. After each passage, samples were taken and plated for single colonies. Three colonies were randomly picked, and the MIC of fluconazole was determined (Table S1D). Derivatives exhibiting increased fluconazole resistance could be isolated after only a few passages in the presence of the drug in all cases. We selected two to four clones from different passages of each original mating product, including the isolates with the highest drug resistance as well as isolates from earlier passages displaying intermediate resistance levels, to investigate the genomic alterations that were associated with resistance development (Fig. 5 and Fig. S5). Remarkably, for most mating products, at least one of the analyzed progeny contained only the mutated form of one or even both of the relevant resistance genes and had lost the corresponding wild-type alleles. The three derivatives of EM1 contained only the mutated ERG11* and MRR1* alleles and had also lost *MTL*α, which was linked to the wild-type *ERG11* in this strain (Fig. 5A). All three derivatives of TM1 had lost the wild-type *TAC1* allele and the linked *MTL*α, and the last isolate with the highest resistance had also lost the wild-type copies of *MRR1* (Fig. 5E). In the derivatives of EU1, sequential loss of the two wild-type *ERG11* alleles (and the linked *MTL*α) was observed, and the relative amount of the mutated *UPC2* (compared to wild-type *UPC2*) was also increased in the last of the two analyzed clones (Fig. 5B). Similarly, the two derivatives of TU1 with the highest drug resistance had lost the wild-type *TAC1* alleles and the linked *MTL*α, and the relative copy number of the mutated UPC2* was increased (Fig. 5F). Progeny of MU1 showed sequential loss of the wild-type *MRR1* alleles (Fig. 5C). Intriguingly, one clone derived from TE1, which showed the highest resistance level in this series, had lost both wild-type *TAC1* alleles but nevertheless retained the mutated ERG11* and *MTL*α (the relative amount of these alleles actually increased compared to TE1), although they were located on the same Chr5 homolog (Fig. 5D). Therefore, a mitotic recombination event that disconnected ERG11* from wild-type *TAC1* must have occurred in this strain to enable the retention of both types of resistance genes (we note that the high resistance of TE1 cannot solely be explained by the loss/decreased copy number of the wild-type alleles, and additional mechanisms must have contributed). Similar events were observed in derivatives of the reciprocal mating products and are documented in Fig. S5. The loss of wild-type alleles in mating product progeny was confirmed by sequencing (Fig. S6), and the observed changes at the relevant genomic loci in all analyzed derivatives of the different mating products are summarized in Table 4.

**TABLE 4:** Genomic changes in mating products after passage in fluconazole

Mating product/progeny	Fluconazole MIC (µg/ml)	Genomic change(s) in mating product progeny^*a*^
EM1	8	
EM1P3A-1	64	Loss of wild-type *MRR1*, wild-type *ERG11*, and *MTL*α
EM1P8A-1	128	Loss of wild-type *MRR1*, wild-type *ERG11*, and *MTL*α
EM1P8A-3	128	Loss of wild-type *MRR1*, wild-type *ERG11*, and *MTL*α
ME1	4	
ME1P4A-2	32	Loss of wild-type *MRR1*
ME1P4A-3	32	Loss of wild-type *MRR1*
ME1P5A-2	64	Loss of wild-type *MRR1*
ET1	8	
ET1P2A-2	16	
ET1P7A-3	32	Loss of wild-type *TAC1-1*, decrease in ERG11* and *MTL***a**
TE1	4	
TE1P6A-1	16	Decrease in wild-type *TAC1*, ERG11*, and *MTL*α
TE1P6A-2	16	Loss of wild-type *TAC1*, ERG11*, and *MTL*α
TE1P12A-1	128	Loss of wild-type *TAC1*, increase in ERG11* and *MTL*α
EU1	4	
EU1P6A-3	32	Decrease in wild-type *ERG11* and *MTL*α
EU1P8A-3	64	Loss of wild-type *ERG11* and *MTL*α, increase in UPC2*
UE1	2	
UE1P2A-1	4	Increase in UPC2*
UE1P4A-3	16	Increase in UPC2*, ERG11*, and *MTL*α
MT1	8	
MT1P3A-3	16	Decrease in wild-type *MRR1*
MT1P5A-2	32	Loss of wild-type *MRR1*
MT1P5A-3	32	Loss of wild-type *MRR1*, wild-type *TAC1*, and *MTL***a**
TM1	4	
TM1P6A-2	16	Loss of wild-type *TAC1* and *MTL*α
TM1P6A-3	16	Loss of wild-type *TAC1* and *MTL*α
TM1P10A-1	64	Loss of wild-type *MRR1*, wild-type *TAC1*, and *MTL*α
MU1	4	
MU1P5A-1	32	Decrease in wild-type *MRR1*
MU1P7A-1	64	Loss of wild-type *MRR1*
UM1	4	
UM1P3A-3	32	Loss of wild-type *MRR1*, increase in UPC2*
UM1P3A-1	64	Loss of wild-type *MRR1*
TU1	4	
TU1P6A-3	16	
TU1P11A-1	64	Loss of wild-type *TAC1* and *MTL*α, increase in UPC2*
TU1P11A-3	64	Loss of wild-type *TAC1* and *MTL*α, increase in UPC2*
UT1	8	
UT1P2A-2	16	Decrease in wild-type *TAC1* and *MTL***a**, increase in UPC2*
UT1P2A-3	16	Decrease in wild-type *TAC1* and *MTL***a**, increase in UPC2*
UT1P5A-1	32	Loss of wild-type *TAC1* and *MTL***a**
UT1P5A-2	32	Decrease in wild-type *TAC1* and *MTL***a**, increase in UPC2*

a Decreases and increases in copy number are relative to the other allele.

10.1128/mBio.02740-18.6 FIG S6 Loss of wild-type alleles in mating product progeny. Download FIG S6, PDF file, 0.2 MB. Copyright © 2019 Popp et al. 2019 Popp et al. This content is distributed under the terms of the Creative Commons Attribution 4.0 International license.

**FIG 6:**
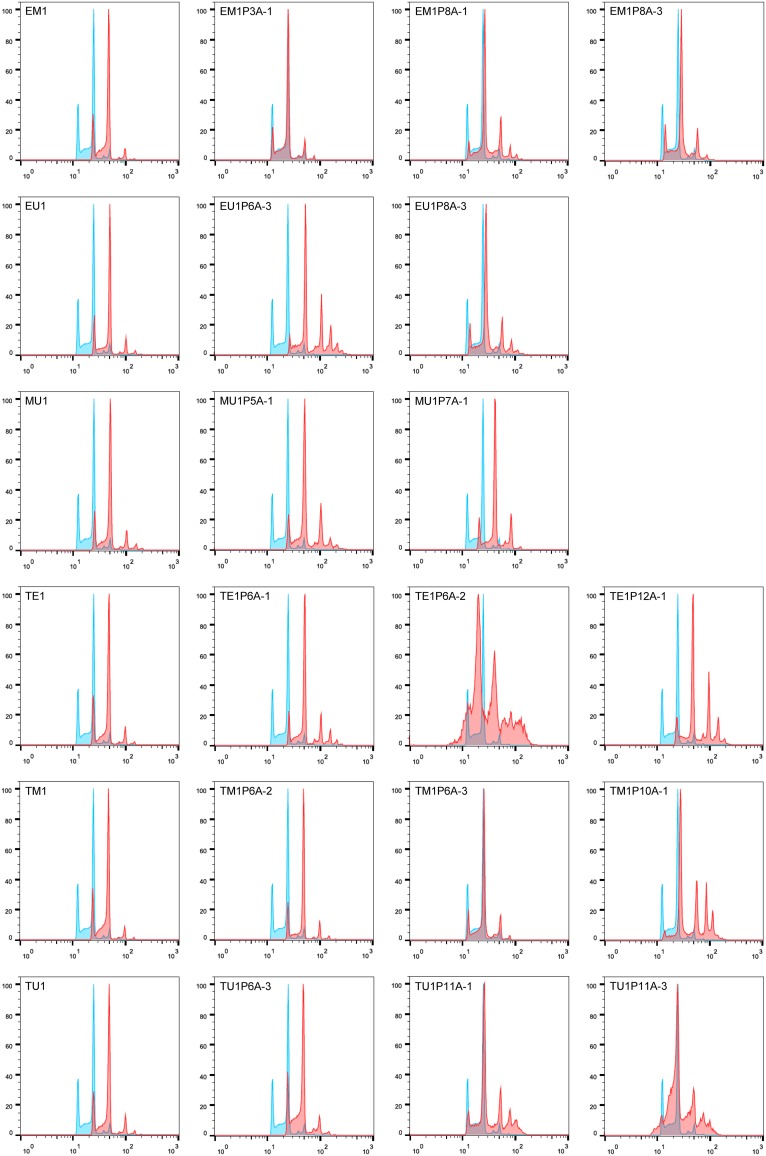
Ploidy analysis of mating product progeny. The plots show the results of flow cytometric measurements of the DNA content of the original mating products and derivatives with increased fluconazole resistance after passage in the presence of the drug. The profile of the diploid reference strain SC5314 is shown in blue in each experiment.

Flow cytometry analysis showed that the ploidy of the mating product progeny had changed in many cases, and some derivatives had returned to the diploid or a near-diploid state, although the cultures also contained subpopulations with variable ploidy levels (Fig. 6 and Fig. S7). The instability of aneuploid strains was also apparent when independent subcultures of the same frozen stock were analyzed (Fig. S8). Altogether, these results demonstrate that tetraploid cells generated by mating of cells with different fluconazole resistance mechanisms can quickly reassort their combined chromosome sets and produce highly drug-resistant progeny that have retained the alleles with resistance mutations.

10.1128/mBio.02740-18.7 FIG S7 Ploidy analysis of mating product progeny. Download FIG S7, PDF file, 0.2 MB. Copyright © 2019 Popp et al. 2019 Popp et al. This content is distributed under the terms of the Creative Commons Attribution 4.0 International license.

10.1128/mBio.02740-18.8 FIG S8 Ploidy analysis of mating product progeny. Download FIG S8, PDF file, 0.8 MB. Copyright © 2019 Popp et al. 2019 Popp et al. This content is distributed under the terms of the Creative Commons Attribution 4.0 International license.

## DISCUSSION

Although *C. albicans* primarily propagates in a clonal fashion, its capacity to mate suggests that a parasexual cycle is used by this fungus to generate diversity (48–50). Mating between unrelated strains, which would result in progeny with new combinations of genetic traits, seems to be rare in *C. albicans*, for the reasons explained in the introduction. Instead, it has been suggested that *C. albicans* undergoes mating between genetically identical or nearly identical cells to produce genetic variation and novel phenotypes (16, 17). Random chromosome loss in tetraploid mating products results in aneuploidies, recombination of homologous chromosomes, and LOH for different parts of the genome at rates that are considerably higher than in diploid cells (16). These genome alterations may increase the fitness of the cells under certain stressful conditions (51), and aneuploid *C. albicans* strains that were derived from tetraploid mating products exhibited wide-ranging differences in fitness and virulence-related properties (17). Here, we have demonstrated that mating between cells in an originally clonal population can also be used by *C. albicans* to combine advantageous traits that have been acquired by individual cells as an adaptation mechanism to a stressful environment. We investigated the development of fluconazole resistance as a case example for a potential involvement of parasexual recombination in adaptive evolution, because resistance to this drug can be acquired by different mechanisms and combinations of these mechanisms, which further increase fluconazole resistance, are frequently observed in highly drug-resistant clinical isolates.

As most *C. albicans* strains are *MTL*-heterozygous, a first prerequisite for cells to combine individually acquired drug resistance mechanisms by parasexual recombination is LOH at the *MTL* locus in order to become mating-competent. LOH, either by mitotic recombination or by transient aneuploidies, can occur spontaneously, but the frequency of such events is strongly increased by various stress conditions, including growth in the presence of fluconazole (44, 45). Although fluconazole-induced LOH does not require the presence of resistance mutations, homozygosity for an existing resistance mutation provides a selective advantage that promotes the enrichment of such cells in the population (42). In our present study, this was most obvious when we screened for *MTL*-homozygous derivatives of strains containing a GOF mutation in one *MRR1* allele after growth in the presence of the drug. Although *MRR1* is not linked to *MTL*, all analyzed *MTL*-homozygous clones were also homozygous for the mutated *MRR1* allele, demonstrating that those *MTL*-homozygous cells that had simultaneously become homozygous for the mutated *MRR1* were favored because of their increased drug resistance. Therefore, in an originally clonal population, those cells that have acquired a resistance mutation will dominate the subpopulation that has become mating-competent under selective pressure by the drug.

Another prerequisite for mating in *C. albicans* is a phenotypic switch of *MTL*-homozygous cells to the opaque morphology (13). This phenotypic transition was for a long time thought to occur stochastically in few cells of a population (52–54), but it is now known that switching to the opaque cell type can be efficiently induced by various environmental conditions that *C. albicans* encounters within its human host (47, 55–57). When we mixed opaque cells of strains with different resistance mechanisms, we readily obtained mating products from all combinations of parental strains even without applying selective conditions. The mating products were unstable, and many had already lost copies of wild-type or mutated alleles when we analyzed subcultures of our frozen stocks. It has been shown that once chromosome loss has started in tetraploid mating products, the cells continue to lose chromosomes to generate progeny with different aneuploidies and may eventually achieve a stable euploid state (16). While the primary mating products, which were heterozygous again for both resistance mutations, did not exhibit higher drug resistance levels than their parental strains, they rapidly developed increased fluconazole resistance under selective pressure by loss of the wild-type alleles. In some cases, the aneuploid state itself may have contributed to the increased drug resistance, as was recently demonstrated for mating product derivatives that did not contain resistance mutations (17). However, it was striking that almost all original mating products generated progeny that had become homozygous for one or even both resistance alleles under selective pressure. Therefore, mating and subsequent reassortment of the combined chromosome sets is an efficient way for *C. albicans* to become highly drug-resistant once individual cells in a population have independently acquired resistance mechanisms with additive effects. Some mating product derivatives had become diploid or near-diploid after the few passages in the presence of fluconazole that were required to select for increased drug resistance, but many were aneuploid. This could reflect the fact that fluconazole induces aneuploidies (45), which in turn may impede the achievement of a stable ploidy level during growth in the presence of the drug. Indeed, many fluconazole-resistant clinical *C. albicans* isolates are aneuploid (8). While this may be a consequence of fluconazole-induced aneuploidy in diploid cells, the drug-resistant clinical isolates could also have arisen by the parasexual cycle, supporting the idea that mating of *C. albicans* could occur within its human host and facilitate adaptation to new adverse conditions.

The combination of drug resistance mechanisms is an illustrative example of how *C. albicans* can harness its hidden mating ability to generate better-adapted genetic variants, but it may apply to other situations in which LOH for a beneficial mutation provides a selective advantage and enriches for *MTL*-homozygous cells. Parasexual recombination, promoted by stress-induced genomic alterations that result in the acquisition of mating competence in cells with adaptive mutations, may therefore be an important mechanism in the evolution of *C. albicans* populations.

## MATERIALS AND METHODS

### Strains and growth conditions.

*C. albicans* strains used in this study are listed in Tables 1 to 4. All strains were stored as frozen stocks with 17.2% glycerol at −80°C. Strains were routinely grown in YPD liquid medium (10 g yeast extract, 20 g peptone, 20 g glucose per liter) at 30°C (white cells) or 25°C (opaque cells) in a shaking incubator. For solid media, 1.5% agar was added before autoclaving. To identify white and opaque colonies, strains were grown at room temperature on Lee’s agar plates, pH 6.8 (58), containing 5 µg/ml phloxine B, which selectively stains opaque colonies pink (59).

### Strain construction.

The inserts from plasmids pERG11R3 (42), pTAC1R3 (60), and pUPC2R1 (31) were used to replace one of the wild-type *ERG11*, *TAC1*, and *UPC2* alleles in strains containing other heterozygous resistance mutations (Table 1) by the *ERG11*^G464S^, *TAC1*^G980E^, and *UPC2*^G648D^ alleles, respectively. The correct integration of each construct was confirmed by Southern hybridization using the flanking sequences as probes. The presence of the mutations was verified by reamplification of the genes and sequencing of the PCR products.

### Southern hybridization analyses.

Genomic DNA from *C. albicans* strains was isolated as described previously (61). The DNA was digested with appropriate restriction enzymes, separated on a 1% agarose gel, transferred by vacuum blotting onto a nylon membrane, and fixed by UV cross-linking. Southern hybridization with enhanced chemiluminescence-labeled probes was performed with the Amersham ECL direct nucleic acid labeling and detection system (GE Healthcare UK Limited, Little Chalfont, Buckinghamshire, United Kingdom) according to the instructions of the manufacturer. The following probes were used for the analysis of specific loci (primer sequences are provided in Table S2 in the supplemental material). For *ERG11*, a 1,488-bp PCR fragment ranging from position +125 to the end of the *ERG11* coding region was amplified with primers ERG4 and ERG9. For *MRR1*, a 3,362-bp fragment covering the complete *MRR1* coding region was amplified with primers ZCF36-1 and ZCF36-2. For *TAC1*, a 1,040-bp PCR fragment ranging from position +1919 to the end of the *TAC1* coding region was amplified with primers TAC1-9 and TAC1-11. For *UPC2*, a 2,158-bp fragment covering the complete *UPC2* coding region was amplified with primers UPC2-1 and UPC2-2. For the *MTL* loci, an 862-bp probe binding in the immediate vicinity of both *MTL***a** and *MTL*α was amplified with primers MTL5F and MTL5R. For *GLN3*, a 2,295-bp fragment comprising the *GLN3* coding region and upstream sequences was amplified with primers GLN1 and GLN5. For *CAP1*, a SacI-BglII fragment from plasmid pCAP1R1 (62) containing *CAP1* sequences from positions +270 to +1000 was used. The binding sites of the probes and the hybridizing fragments are illustrated in Fig. S2.

10.1128/mBio.02740-18.10 TABLE S2 Primers used in this study. Download Table S2, XLSX file, 0.01 MB. Copyright © 2019 Popp et al. 2019 Popp et al. This content is distributed under the terms of the Creative Commons Attribution 4.0 International license.

### Fluconazole MIC assays.

The fluconazole susceptibilities of the strains were determined by a previously described broth microdilution method (63), with slight modifications. A 2-day-old colony from a YPD agar plate was suspended in 2 ml of an 0.9% NaCl solution, and 4 µl of the suspension was mixed with 2 ml 2× SD-CSM medium (13.4 g yeast nitrogen base without amino acids [YNB; MP Biomedicals, Illkirch, France], 40 g glucose, 1.58 g complete supplement medium [MP Biomedicals] per liter). A 2-fold dilution series of fluconazole (Sigma GmbH, Deisenhofen, Germany) was prepared in water, starting from an initial concentration of 512 µg/ml. One hundred microliters of each fluconazole solution was then mixed with 100 µl of the cell suspension in a 96-well microtiter plate, and the plates were incubated for 48 h at 37°C. The MIC of fluconazole was defined as the drug concentration that abolished or drastically reduced visible growth compared to a drug-free control.

### Dilution spot assays.

Overnight cultures of the strains in YPD medium were diluted to an optical density at 600 nm of 2.0. Tenfold dilutions from 10^0^ to 10^−5^ were prepared in a 96-well microtiter plate, and ca. 5 µl of the cell suspensions was transferred with a replicator onto YPD agar plates without or with 5 µg/ml fluconazole. Plates were incubated for 2 days at 30°C and photographed.

### Isolation of fluconazole-induced *MTL*-homozygous strains.

YPD overnight cultures of the strains that were heterozygous for specific resistance mutations were diluted 1:100 in fresh YPD medium with fluconazole and grown for 1 day at 30°C. The cultures were then subcultivated daily for up to eight times by diluting them 1:100 in fresh medium with the same fluconazole concentration (see Table S1A). Appropriate dilutions of each subculture were plated for single colonies on YPD plates with 5 µg/ml fluconazole, to screen for clones with increased drug resistance, or on Lee’s agar plates with phloxine B, to screen for clones that were able to switch to the opaque phase, as explained in Results. The fluconazole concentrations were arbitrarily chosen based on the MICs for the starting strains (Table 1). To screen for *TAC1*-homozygous clones, a fluconazole concentration of 5 µg/ml was used. For *ERG11* and *UPC2*, a lower concentration (2.5 µg/ml) was used, because the mutations in these genes have a weaker effect on fluconazole resistance than the *TAC1* mutation (Table 1). As no *MTL*-homozygous clones that were also homozygous for the *UPC2* mutation were obtained, 5 µg/ml fluconazole was additionally used for these strains. For the *MRR1* strains, 5 µg/ml fluconazole was initially used, but no *MTL*-homozygous clones were obtained in this experiment. Therefore, two lower fluconazole concentrations (2.5 µg/ml and 1.75 µg/ml) were used in the next experiment, which resulted in the successful isolation of six *MTL*-homozygous clones. No efforts were made to further optimize the conditions for the selection of *MTL*-homozygous strains.

### Mating experiments.

Opaque cells of the *MTL*-homozygous strains were freshly streaked from frozen stocks onto Lee’s agar plates with phloxine B and incubated for several days at room temperature. An opaque colony of each strain was inoculated into YPD medium and grown overnight at 25°C. *MTL***a** and *MTL*α strains were mixed in equal proportions (3 × 10^7^ cells of each strain) and spotted on Spider agar plates (1% nutrient broth, 2% mannitol, 0.4% dipotassium phosphate, 1.35% agar, pH 7.2). To prevent spontaneous switching of opaque cells to the white phase, the plates were first incubated for 2 days in a CO_2_ incubator at 18% CO_2_, 25°C, and then further incubated in a normal atmosphere at room temperature for up to 12 days. At various times, samples were taken from different regions of the cell lawn, suspended in water, and plated for single colonies on Lee’s agar with phloxine B. In some cases (UT and EM crossings), the cells were suspended in phosphate-buffered saline and sonicated (Bandelin, Sonopuls, HD 70) to disperse cell clumps, but this step was later omitted because it was found to be unnecessary. The plates were incubated for 2 days at 18% CO_2_, 25°C, to maintain unmated cells in the opaque phase, followed by incubation for 5 days at room temperature in a normal atmosphere. White colonies were picked and used for genetic analysis. Frozen stocks of the candidate mating products were prepared from an overnight culture of the originally isolated colony.

### Isolation of mating product progeny with increased fluconazole resistance.

A single colony of each selected mating product was taken from a YPD agar plate and grown for 24 h in YPD medium with fluconazole at 30°C. The cultures were diluted 1:1,000 in fresh medium and grown again for 24 h at 30°C. This step was repeated until passage 12 was reached. The concentration of fluconazole in the first culture was half the MIC for the original mating product and was doubled on every second passage. An aliquot of each culture was mixed with 17.2% glycerol and stored at −80°C. If necessary, subcultures were started from the glycerol stocks. In parallel, subcultures were appropriately diluted, either directly or from the frozen stocks, and spread for single colonies on a YPD agar plate. After 2 days of growth at 30°C, three colonies were randomly picked and used to determine the MIC of fluconazole and to prepare frozen stocks of the individual clones.

### Flow cytometry.

Ploidy measurements were performed similarly to a previously described protocol (64), with some modifications. Strains were grown overnight in a 96-well microtiter plate at 30°C in YPD medium and subcultured in fresh YPD medium for 4 h at 30°C. The cells were pelleted, resuspended in 20 µl of 50:50 TE (50 mM Tris-Cl, pH 8; 50 mM EDTA), and fixed with 90% ethanol overnight at −20°C. The cells were then washed twice with 50:50 TE, resuspended in 50 µl RNase A solution (1 mg/ml RNase A in 50:50 TE), and incubated for 3 h at 37°C. The cells were centrifuged, resuspended in 50 µl proteinase K solution (2.5 mg/ml proteinase K [Carl Roth GmbH + Co., Karlsruhe, Germany] in 50:50 TE), and incubated for 30 min at 37°C. After pelleting and resuspending the cells in 50 µl 50:50 TE, 50 µl SYBR Green solution (SYBR Green I [Invitrogen, USA] diluted 1:100 in 50:50 TE) was added and the cells were incubated overnight at room temperature. The cells were washed twice with 50:50 TE and analyzed by flow cytometry with a MACSQuantAnalyzer (Miltenyi Biotec, Bergisch Gladbach, Germany). Data of at least 10,000 cells were collected and analyzed with FlowJo 10 (Becton, Dickinson and Company, USA).

### DNA sequence analysis.

To verify the presence or absence of wild-type and mutated alleles in mating products and their progeny, relevant parts of these genes were amplified from genomic DNA of the strains and sequenced (Microsynth Seqlab, Göttingen, Germany). *ERG11* was amplified with primers ERG9 + ERG2 and sequenced with primer ERG2. *MRR1* was amplified with primers ZCF36seq2 + ZCF36seq5 and sequenced with primer ZCF36seq3. *TAC1* was amplified with primers TAC1-12 + TAC1-7 and sequenced with primer TAC1-12. *UPC2* was amplified with primers UPC2-1 + UPC2-2 and sequenced with primer UPC2-2.
