# Supplementary material for: Evolution of Fluconazole-Resistant Candida albicans Strains by Drug-Induced Mating Competence and Parasexual Recombination
Source: mBio. 2019 Feb 5;10(1):e02740-18. doi: 10.1128/mBio.02740-18 (PMC6428756; doi:10.1128/mBio.02740-18)
Supplement: FIG S1 [file mBio.02740-18-sf001.pdf]

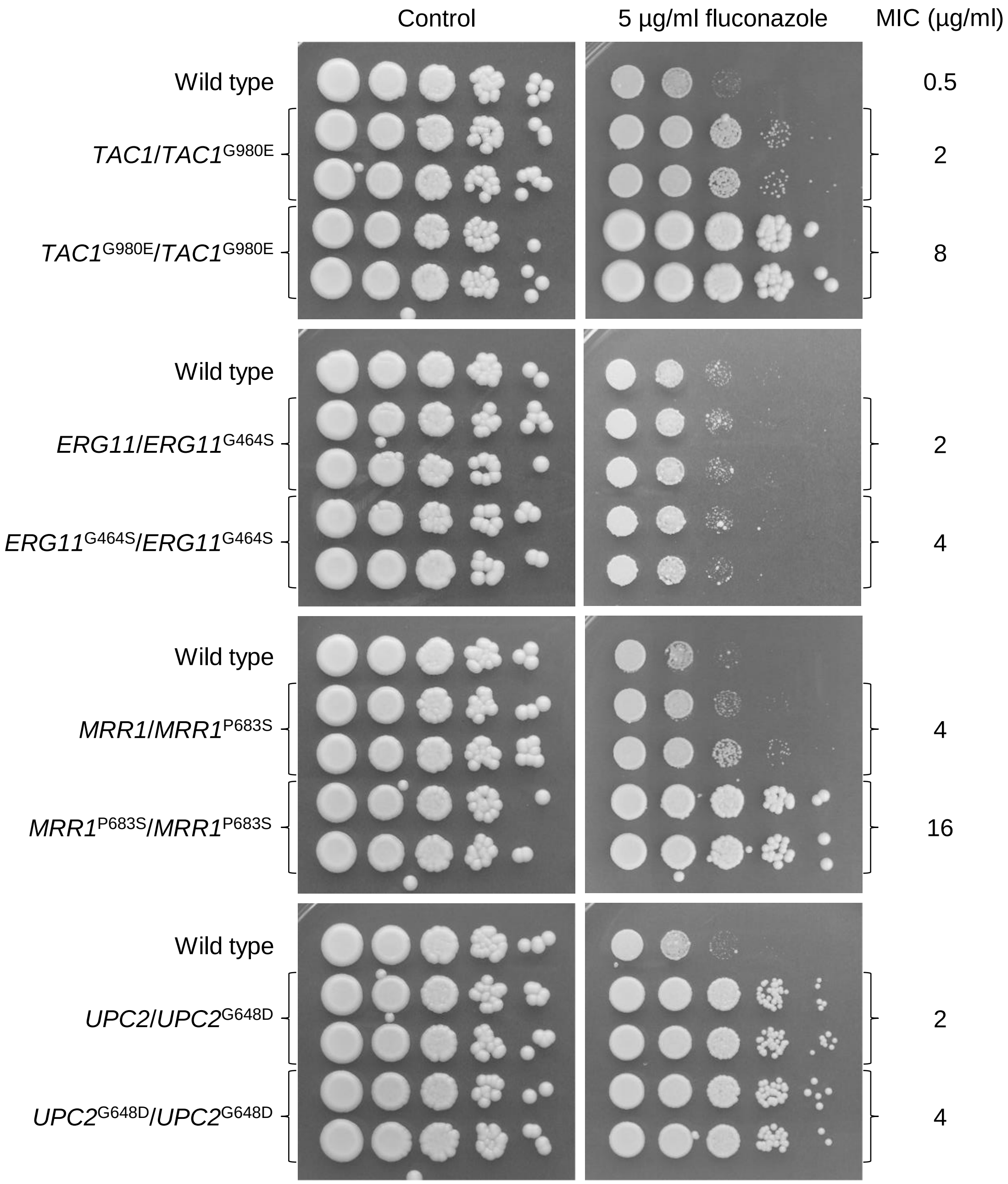

Fig S1. Growth of strains with heterozygous and homozygous resistance mutations on agar plates containing fluconazole. Serial 10-fold dilutions of the strains were spotted on YPD agar plates without or with 5 µg/ml fluconazole and incubated for 2 days at 30°C. The following strains were used: SC5314 (wild type), SCTAC1R32A and -B (*TAC1*/*TAC1*^{G980E}), SCTAC1R34A and -B (*TAC1*^{G980E}/*TAC1*^{G980E}), SCERG11R32A and -B (*ERG11*/*ERG11*^{G464S}), SCERG11R34A and -B (*ERG11*^{G464S}/*ERG11*^{G464S}), SCMRR1R32A and -B (*MRR1*/*MRR1*^{P683S}), SCMRR1R34A and -B (*MRR1*^{P683S}/*MRR1*^{P683S}), SCUPC2R12A and -B (*UPC2*/*UPC2*^{G648D}), SCUPC2R14A and -B (*UPC2*^{G648D}/*UPC2*^{G648D}). The MICs of fluconazole for each strain, as determined in a broth microdilution assay, are also given.
