# Supplementary material for: Evolution of Fluconazole-Resistant Candida albicans Strains by Drug-Induced Mating Competence and Parasexual Recombination
Source: mBio. 2019 Feb 5;10(1):e02740-18. doi: 10.1128/mBio.02740-18 (PMC6428756; doi:10.1128/mBio.02740-18)
Supplement: FIG S2 [file mBio.02740-18-sf002.pdf]

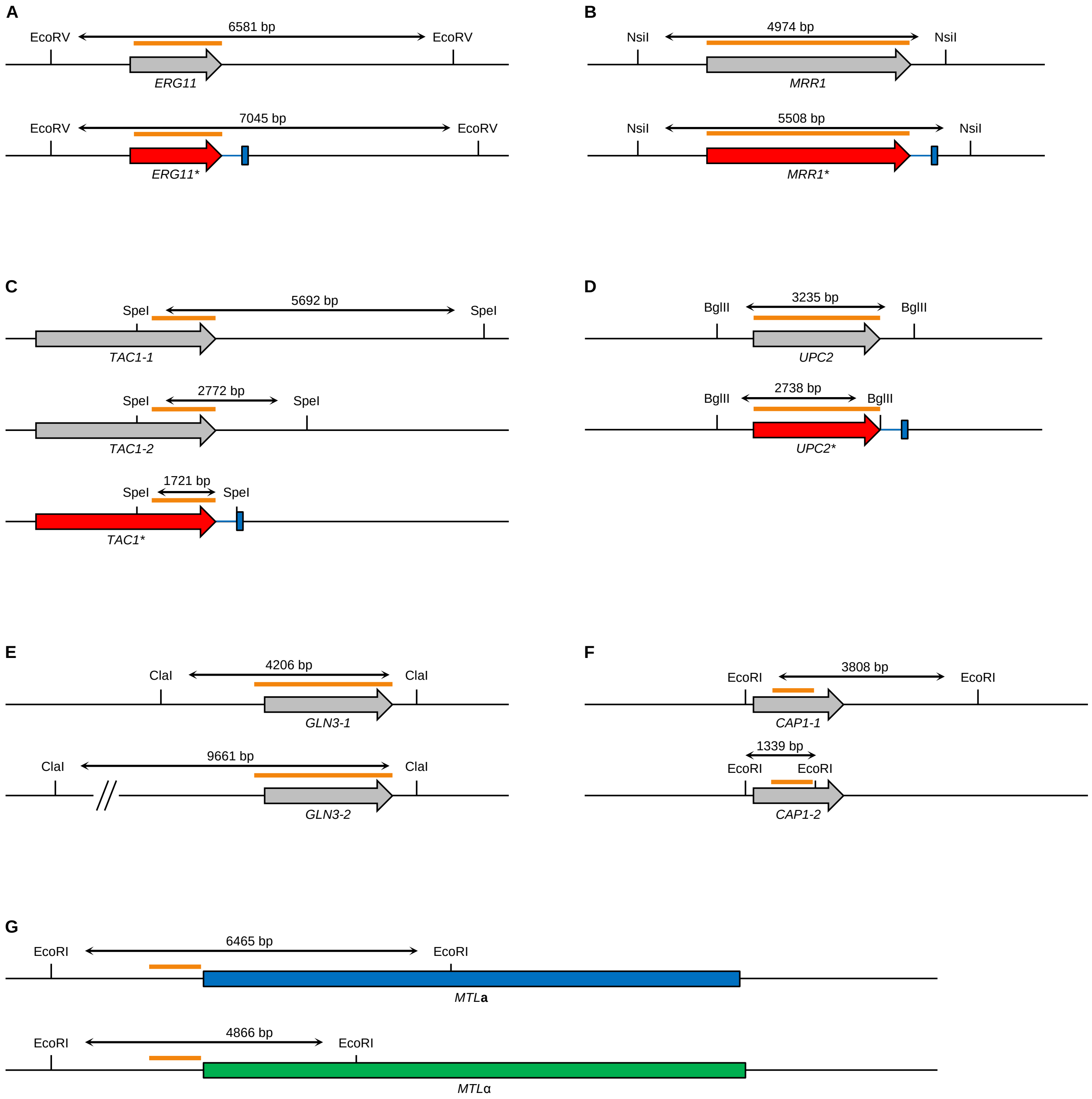

Fig S2. Discrimination between wild-type and mutated *ERG11*, *MRR1*, *TAC1*, and *UPC2* alleles and between the polymorphic wild-type *GLN3*, *CAP1*, and *MTL* alleles in derivatives of strain SC5314 by Southern hybridization. Relevant restriction sites are indicated and the sizes of fragments hybridizing to specific probes (orange bars) are given. The blue line and box behind *ERG11**, *MRR1**, *TAC1**, and *UPC2** indicate the *ACT1* transcription termination sequence and the *FRT* site, respectively, that were introduced together with the mutated alleles (highlighted in red) during strain construction. The blue and green bars in (G) represent the region that differs between the *MTL***a** and *MTL*α loci.
