# Supplementary material for: Evolution of Fluconazole-Resistant Candida albicans Strains by Drug-Induced Mating Competence and Parasexual Recombination
Source: mBio. 2019 Feb 5;10(1):e02740-18. doi: 10.1128/mBio.02740-18 (PMC6428756; doi:10.1128/mBio.02740-18)
Supplement: FIG S3 [file mBio.02740-18-sf003.pdf]

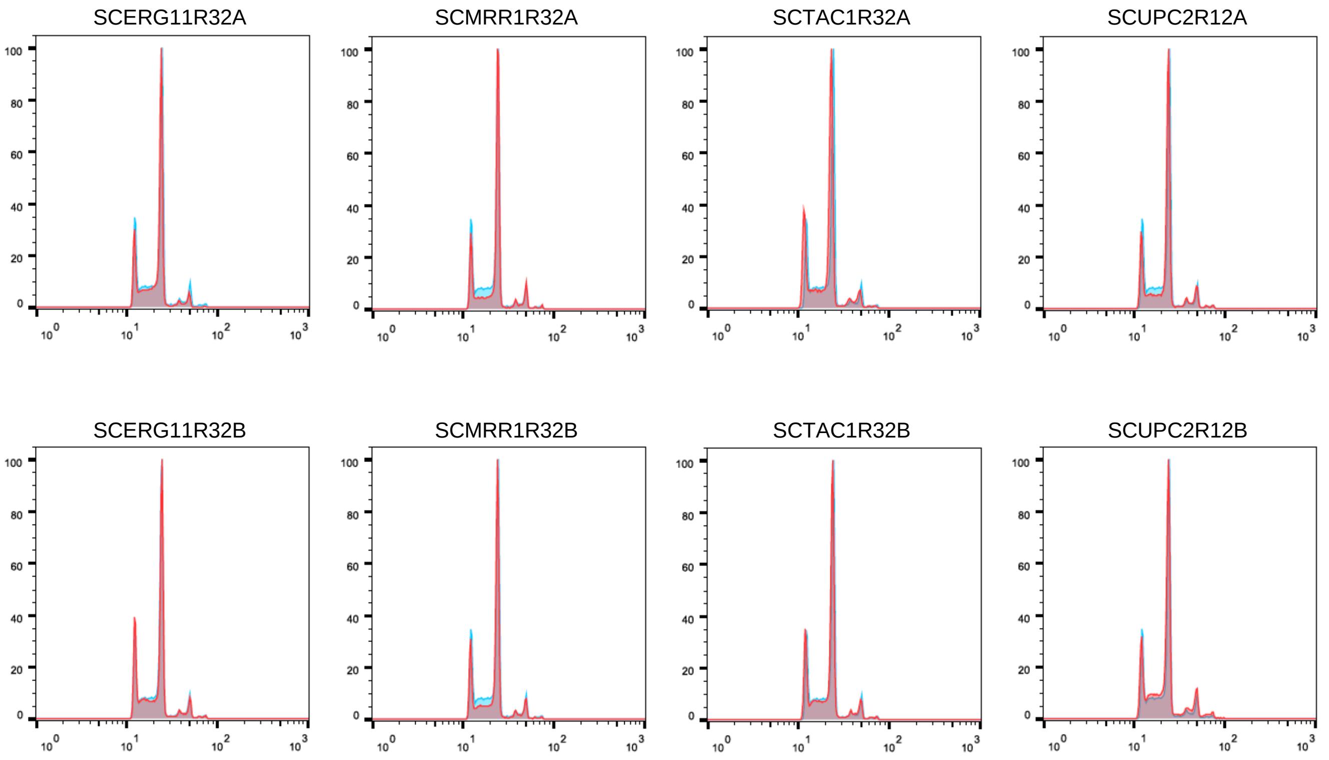

Fig S3. Ploidy analysis of *C. albicans* strains containing different fluconazole resistance mutations. The plots show the results of flow cytometric measurements of the DNA content of strains SCERG11R32A and -B (*ERG11*/*ERG11*^{G464S}), SCMRR1R32A and -B (*MRR1*/*MRR1*^{P683S}), SCTAC1R32A and -B (*TAC1*/*TAC1*^{G980E}), and SCUPC2R12A and -B (*UPC2*/*UPC2*^{G648D}). The profile of the diploid reference strain SC5314 is shown in blue, and the profiles of all other strains are shown in red.
