# Supplementary material for: Evolution of Fluconazole-Resistant Candida albicans Strains by Drug-Induced Mating Competence and Parasexual Recombination
Source: mBio. 2019 Feb 5;10(1):e02740-18. doi: 10.1128/mBio.02740-18 (PMC6428756; doi:10.1128/mBio.02740-18)
Supplement: FIG S4 [file mBio.02740-18-sf004.pdf]

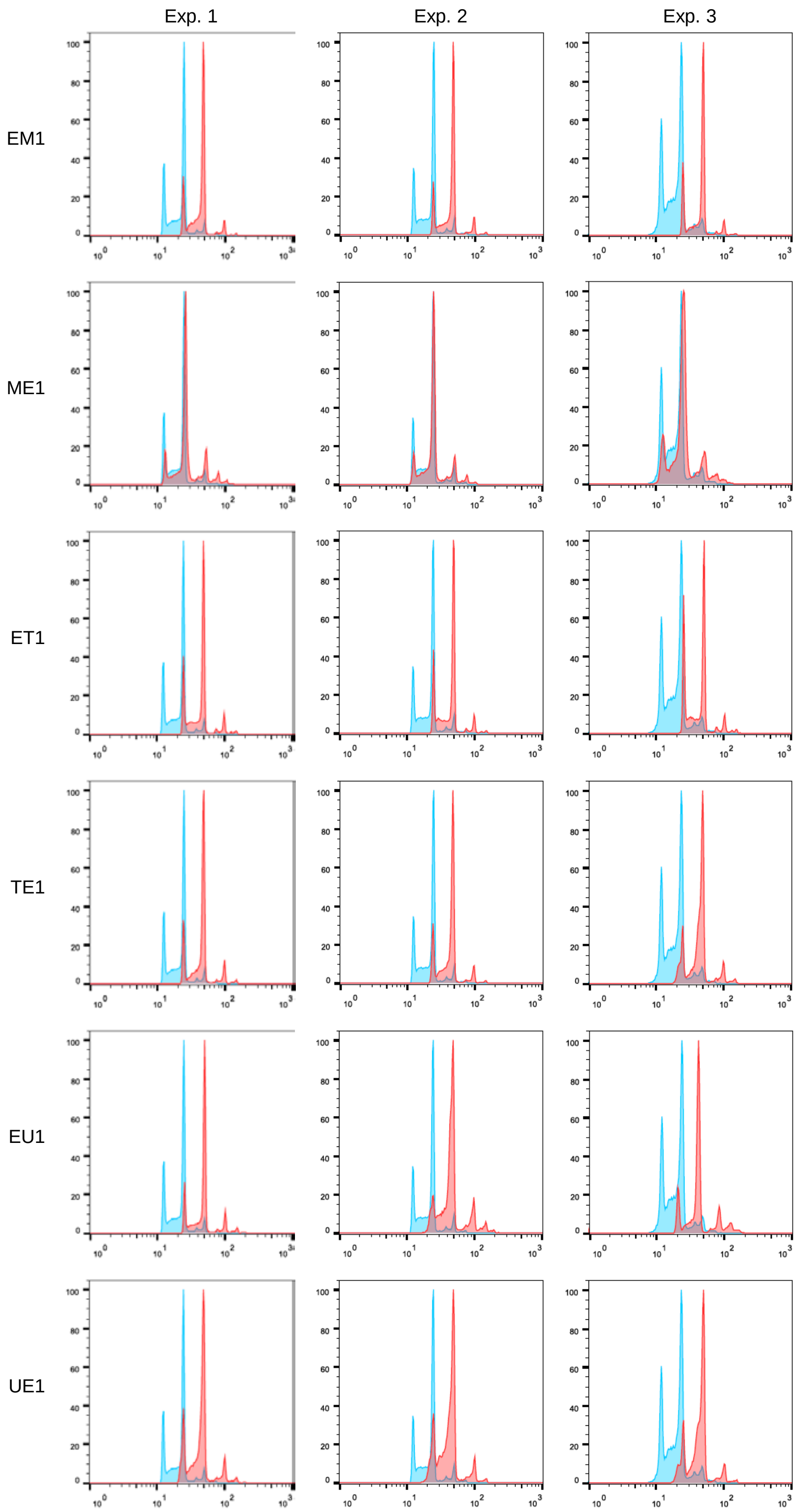

Fig S4. Ploidy analysis of mating products. The plots show the results of three independent flow cytometric measurements of the DNA content of the different mating products. The profile of the diploid reference strain SC5314 is shown in blue in each experiment.

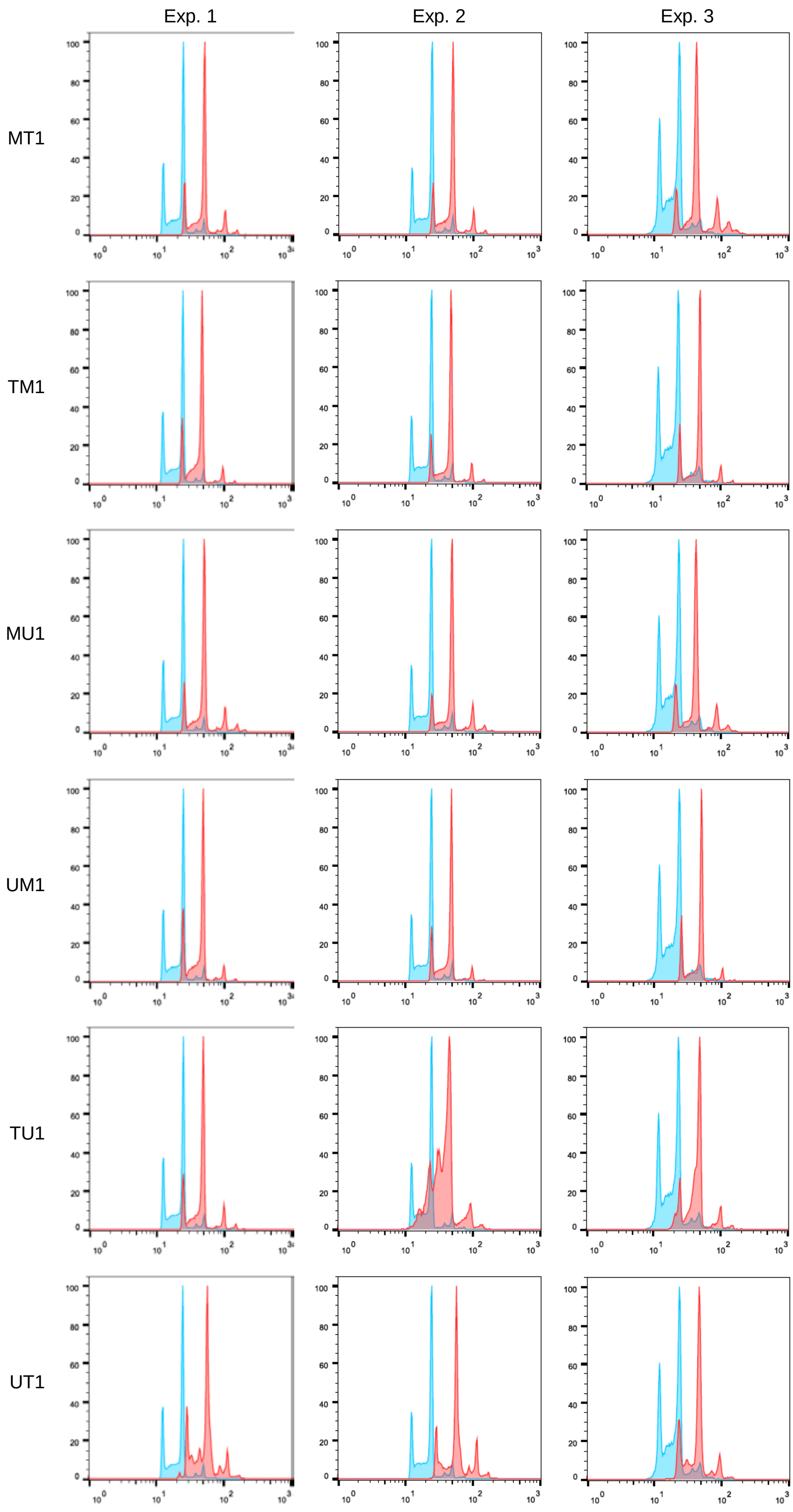

Fig S4 continued.
