# Supplementary material for: Evolution of Fluconazole-Resistant Candida albicans Strains by Drug-Induced Mating Competence and Parasexual Recombination
Source: mBio. 2019 Feb 5;10(1):e02740-18. doi: 10.1128/mBio.02740-18 (PMC6428756; doi:10.1128/mBio.02740-18)
Supplement: FIG S5 [file mBio.02740-18-sf005.pdf]

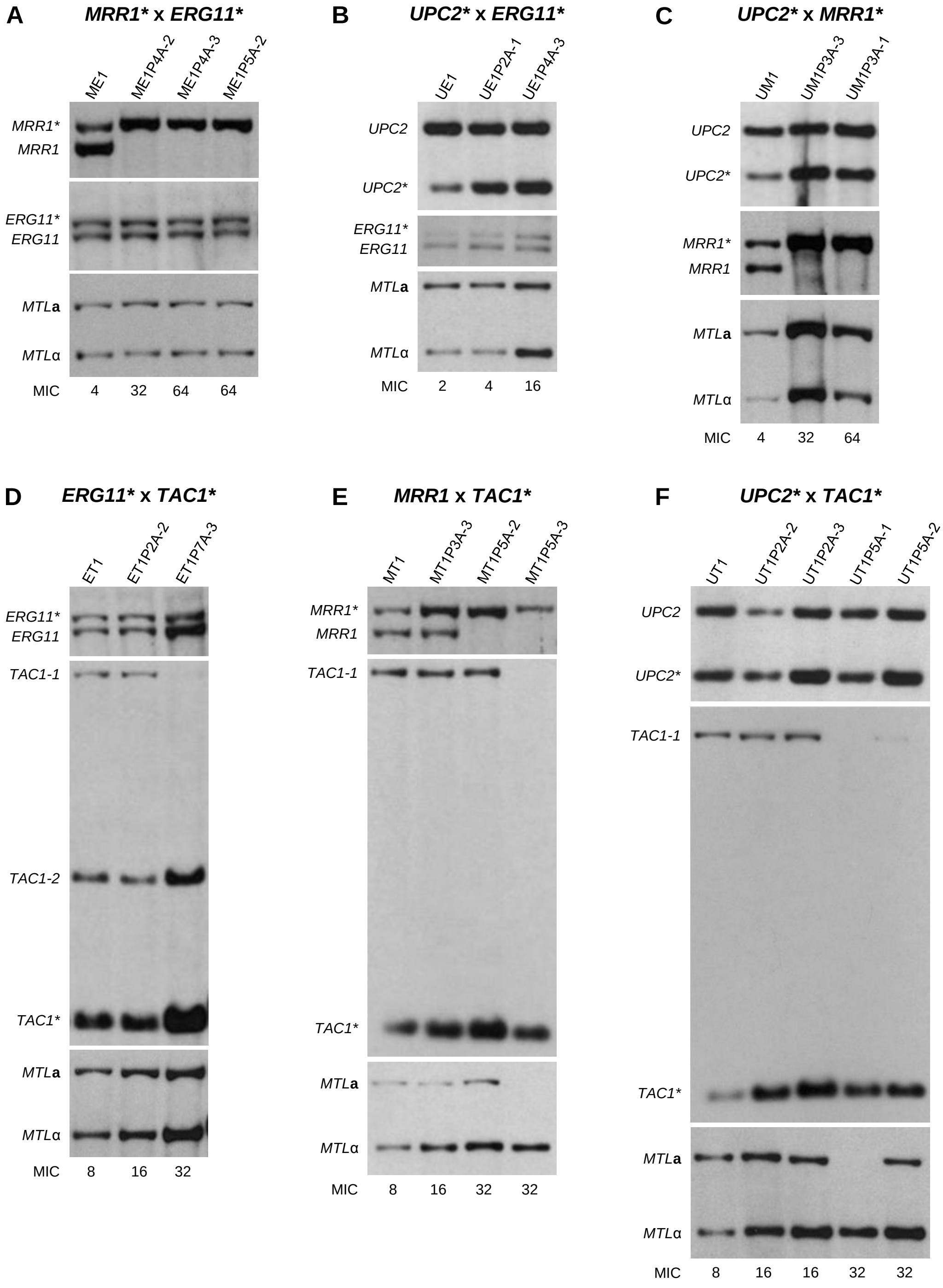

Fig S5. Genetic analysis of mating product progeny by Southern hybridization with relevant probes. Shown are original mating products from reverse crosses of those presented in Fig 5 (first lanes) and derivatives exhibiting increased fluconazole resistance after passage in the presence of the drug. Some blots are from independent genomic DNA preparations of the same strains, and differences in signal intensities are caused by unequal loading. The MIC of fluconazole for each strain is given below the corresponding lane. The observed genetic changes are summarized in Table 4.
