# Supplementary material for: Evolution of Fluconazole-Resistant Candida albicans Strains by Drug-Induced Mating Competence and Parasexual Recombination
Source: mBio. 2019 Feb 5;10(1):e02740-18. doi: 10.1128/mBio.02740-18 (PMC6428756; doi:10.1128/mBio.02740-18)
Supplement: FIG S6 [file mBio.02740-18-sf006.pdf]

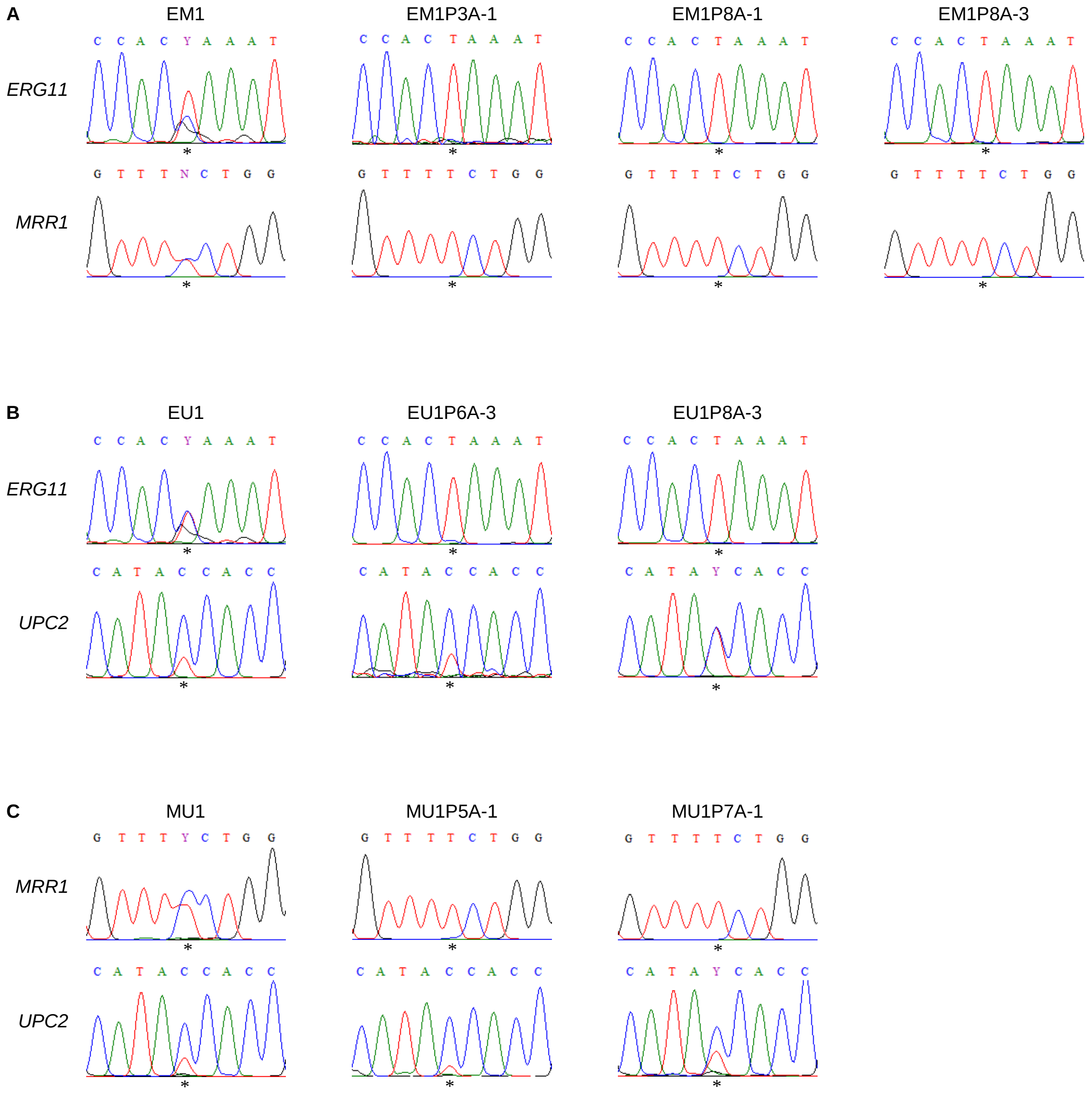

Fig S6. Loss of wild-type alleles in mating product progeny. The *ERG11*, *MRR1*, *TAC1*, and *UPC2* alleles were amplified from genomic DNA of mating products and derivatives with increased fluconazole resistance and sequenced. The images show the regions containing fluconazole resistance mutations (marked by stars *): G1390A (G464S) in *ERG11*, C2047T (P683S) in *MRR1*, G2939A (G980E) in *TAC1*, G1943A (G648D) in *UPC2*. For *ERG11* and *UPC2*, the antisense strand was sequenced. (A) Loss of the wild-type *ERG11* and *MRR1* alleles in derivatives of EM1. (B) Loss of wild-type *ERG11* and increase in *UPC2** in EU1P8A-3. (C) Loss of wild-type *MRR1* in derivatives of MU1. (D) Loss of wild-type *TAC1* and retention of *ERG11** in TE1P12A-1. (E) Loss of wild-type *TAC1* in derivatives of TM1 and loss of wild-type *MRR1* in TMP10A-1. (F) Loss of wild-type *TAC1* and increase in *UPC2** in derivatives of TU1. (G) Loss of wild-type *MRR1* in derivatives of ME1. (H) Increase in *UPC2** and *ERG11** in derivatives of UE1. (I) Loss of wild-type *MRR1* and increase in *UPC2** in derivatives of UM1 (K) Loss of one wild-type *TAC1* allele and decrease in *ERG11** in ET1P7A-3. (L) Sequential loss of the wild-type *MRR1* and *TAC1* alleles in derivatives of MT1. (M) Loss of wild-type *TAC1* in UT1P5A-1.

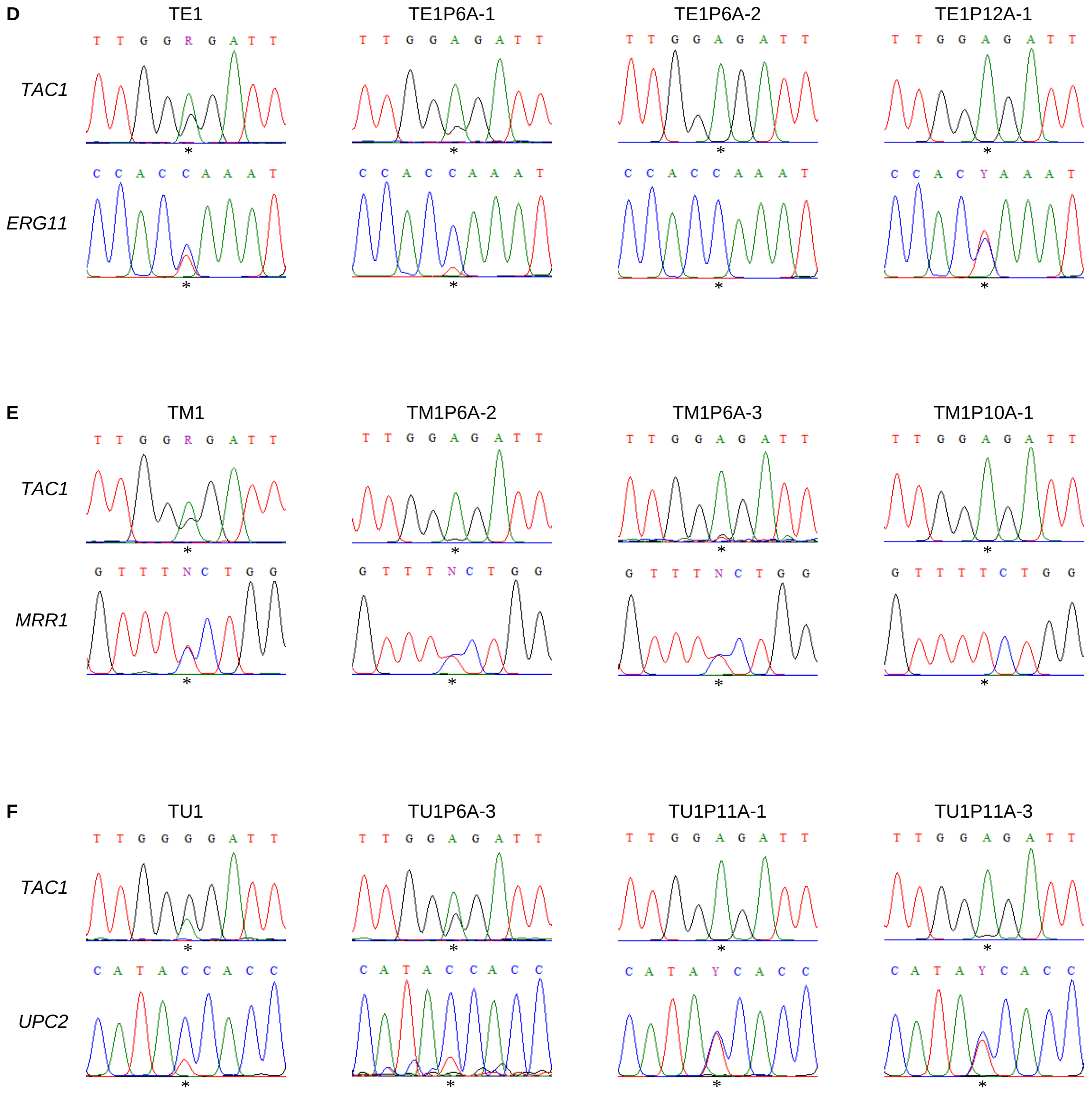

Fig S6 continued.

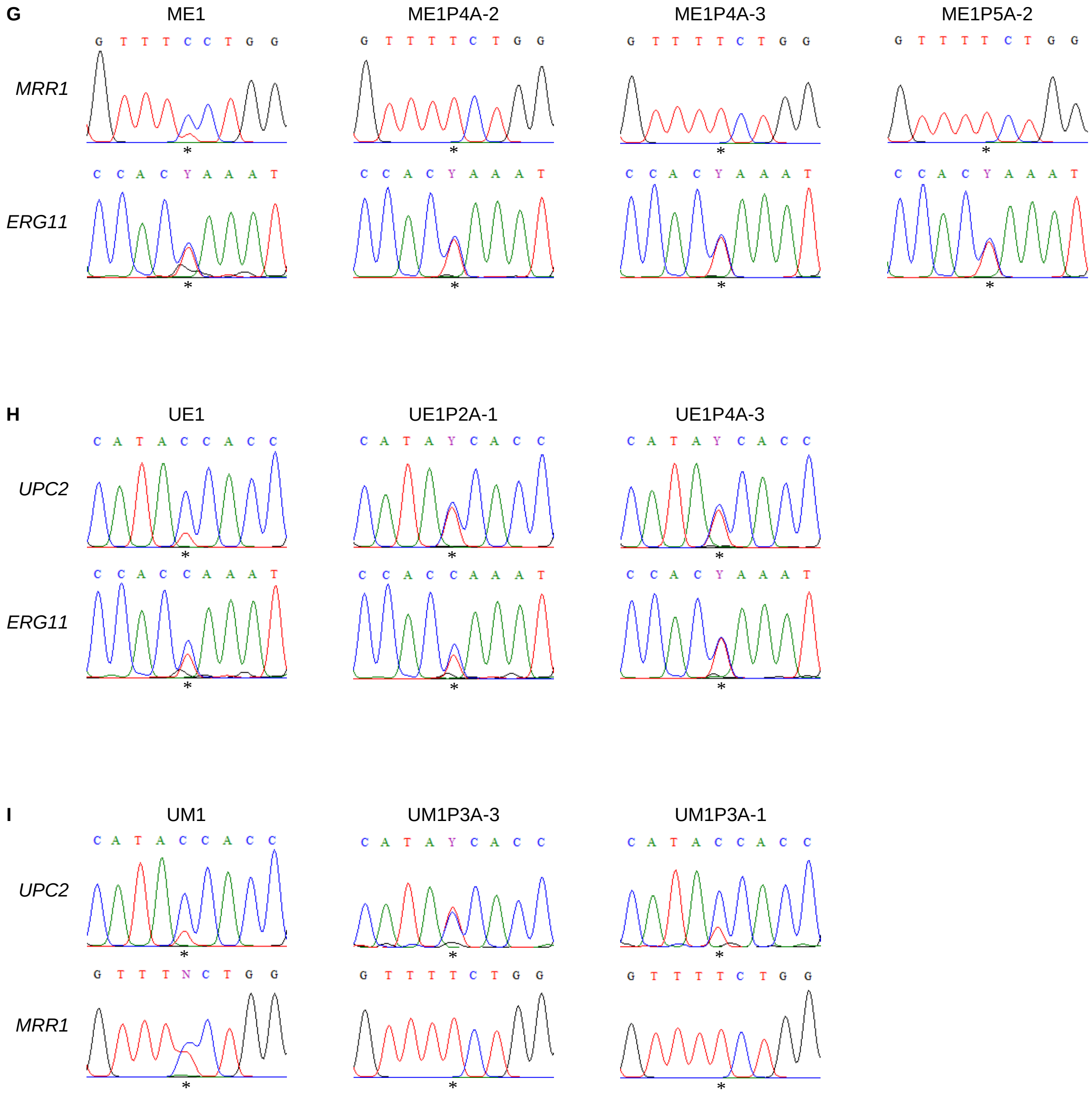

Fig S6 continued.

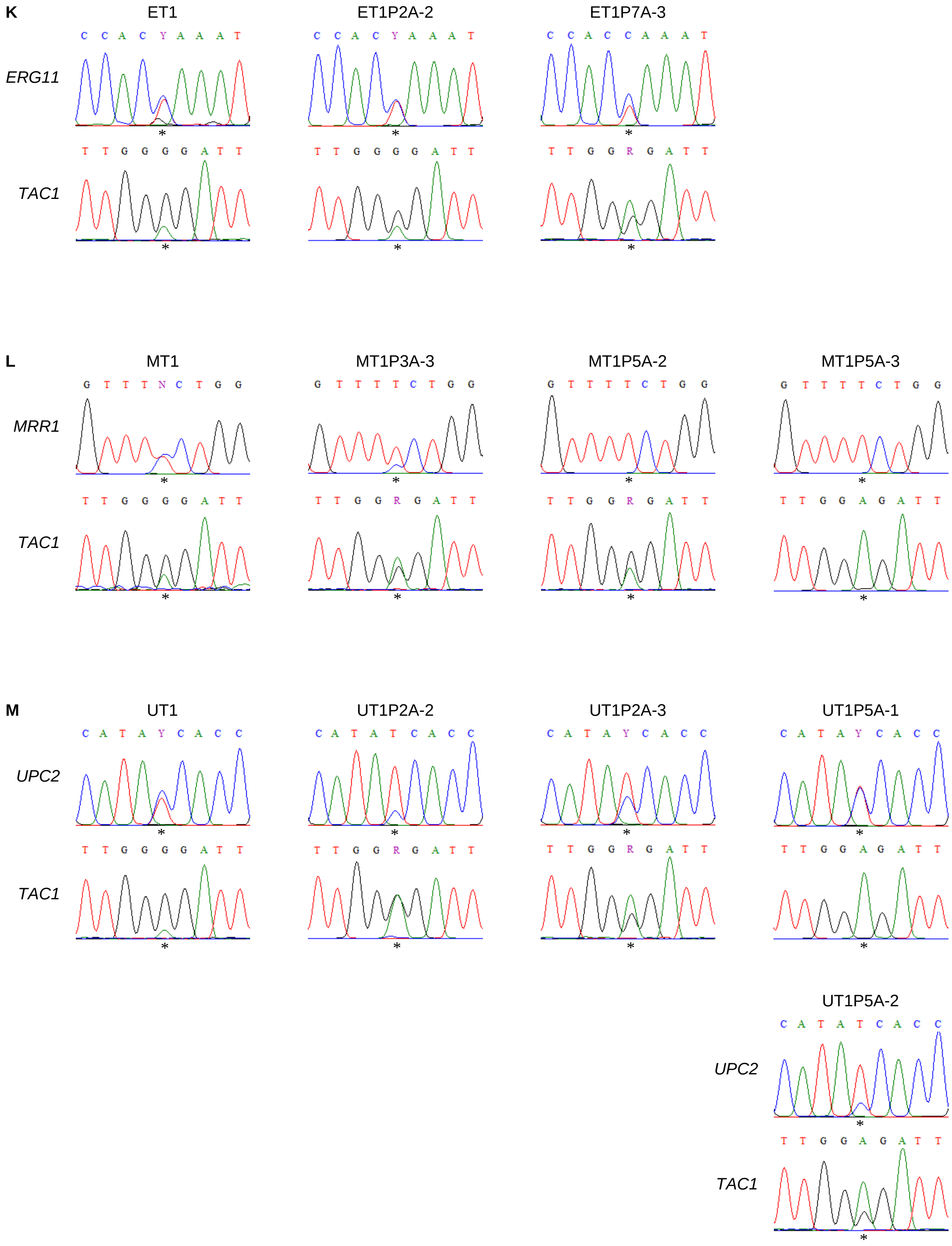

Fig S6 continued.
