# Supplementary material for: Evolution of Fluconazole-Resistant Candida albicans Strains by Drug-Induced Mating Competence and Parasexual Recombination
Source: mBio. 2019 Feb 5;10(1):e02740-18. doi: 10.1128/mBio.02740-18 (PMC6428756; doi:10.1128/mBio.02740-18)
Supplement: FIG S7 [file mBio.02740-18-sf007.pdf]

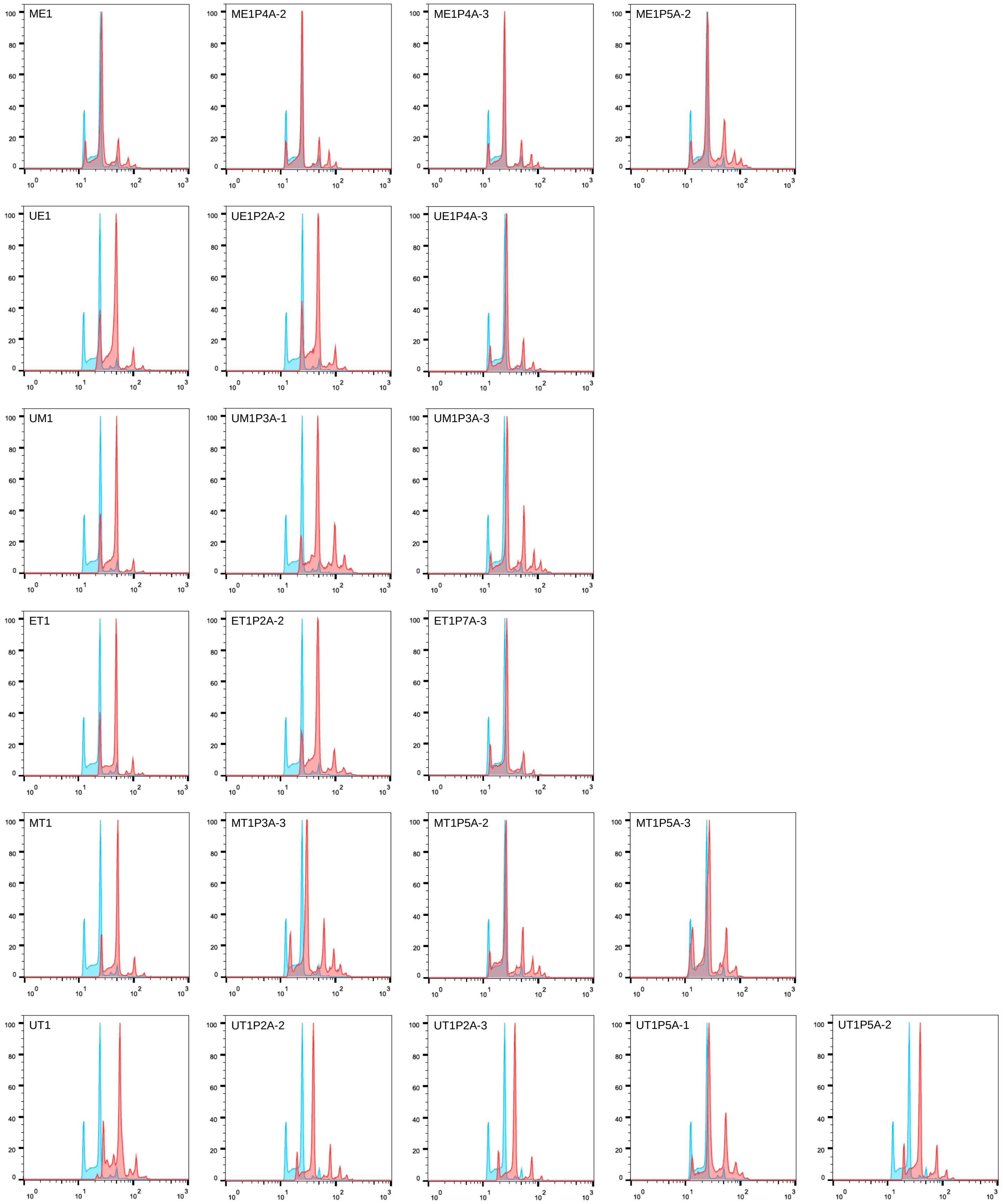

Fig S7. Ploidy analysis of mating product progeny. The plots show the results of flow cytometric measurements of the DNA content of the original mating products from reverse crosses of those presented in Fig 6 and derivatives with increased fluconazole resistance after passage in the presence of the drug. The profile of the diploid reference strain SC5314 is shown in blue in each experiment.
