# Supplementary material for: Evolution of Fluconazole-Resistant Candida albicans Strains by Drug-Induced Mating Competence and Parasexual Recombination
Source: mBio. 2019 Feb 5;10(1):e02740-18. doi: 10.1128/mBio.02740-18 (PMC6428756; doi:10.1128/mBio.02740-18)
Supplement: FIG S8 [file mBio.02740-18-sf008.pdf]

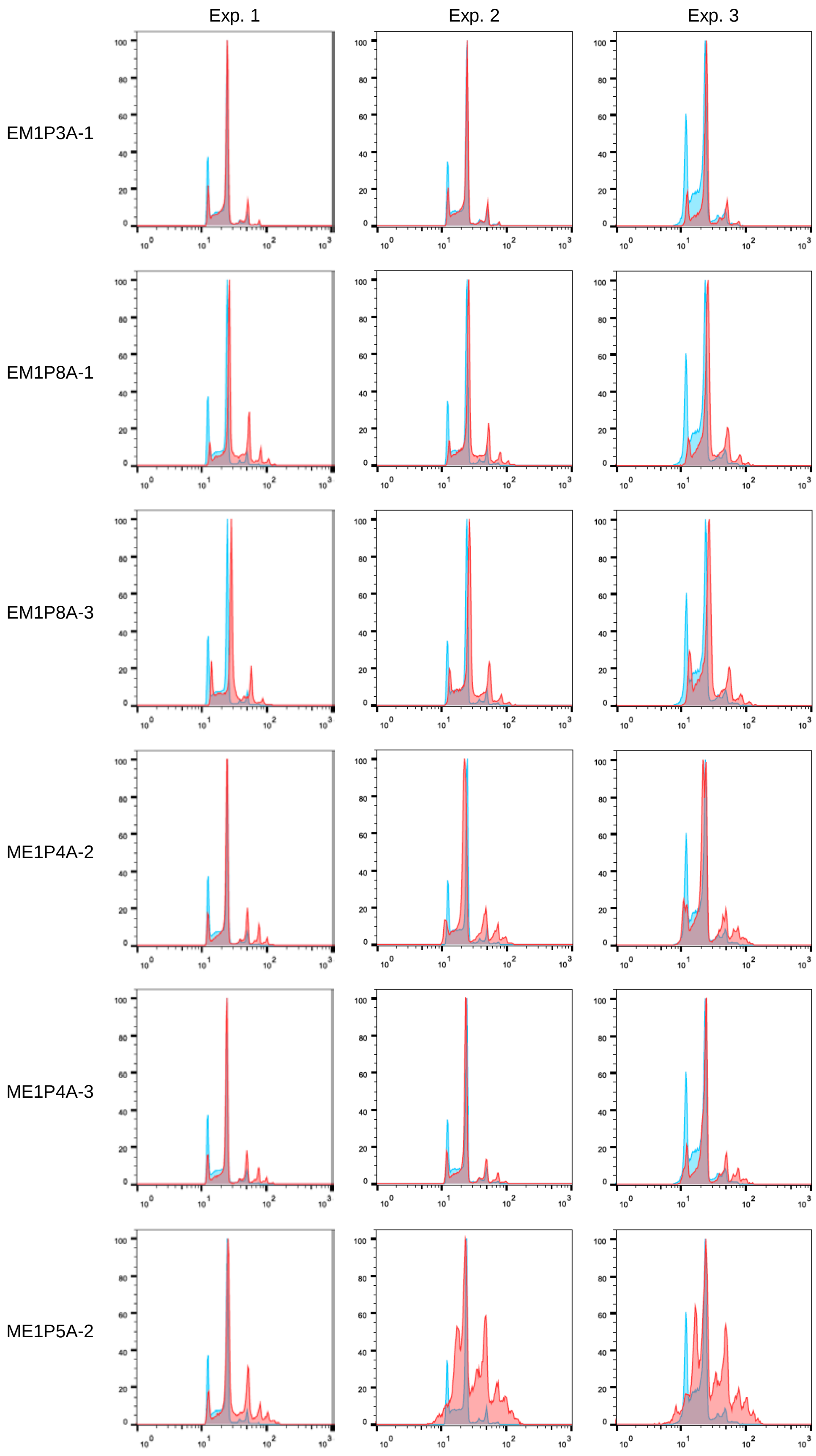

Fig S8. Ploidy analysis of mating product progeny. The plots show the results of three independent flow cytometric measurements of the DNA content of mating product derivatives with increased fluconazole resistance after passage in the presence of the drug. The profile of the diploid reference strain SC5314 is shown in blue in each experiment.

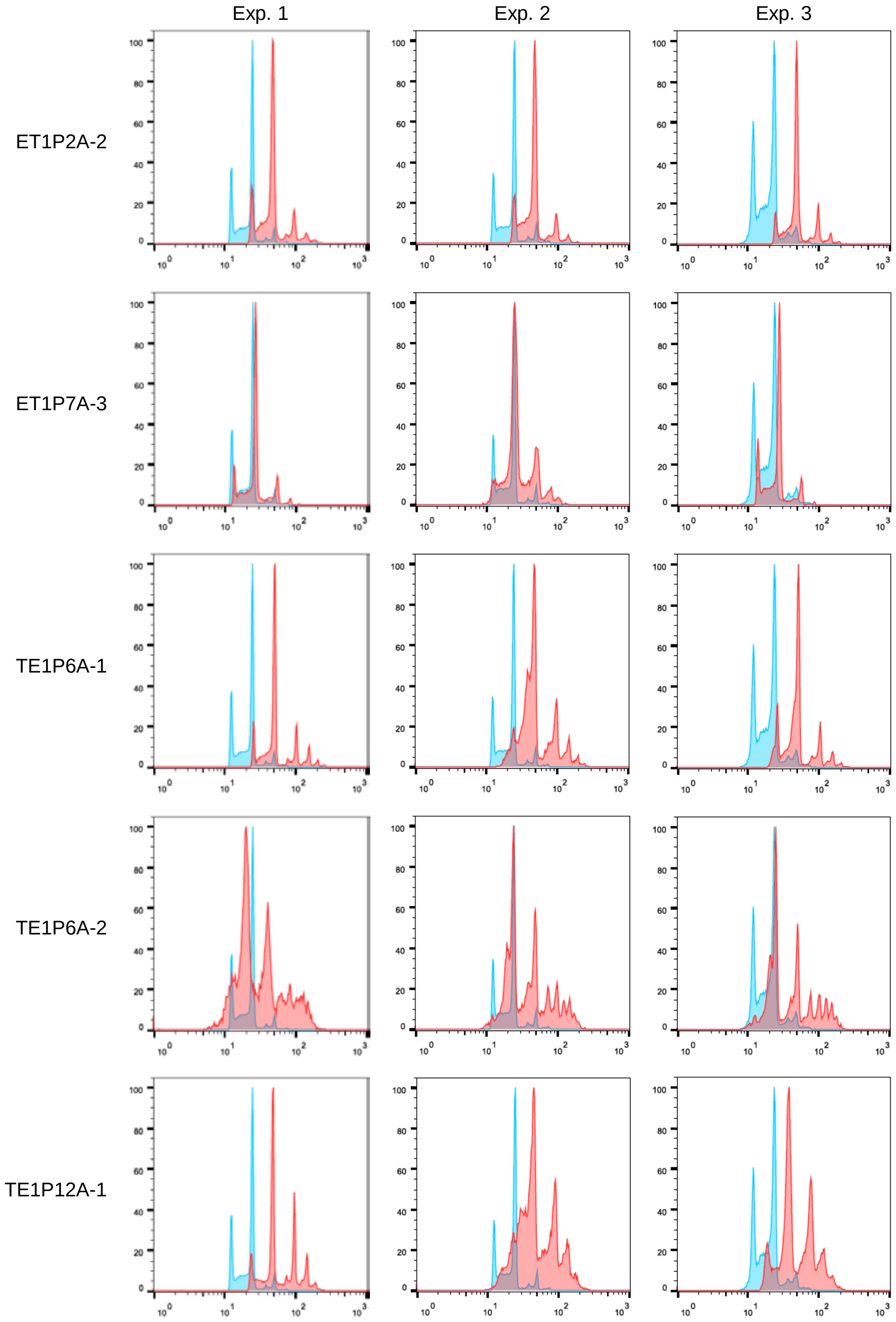

Fig S8 continued.

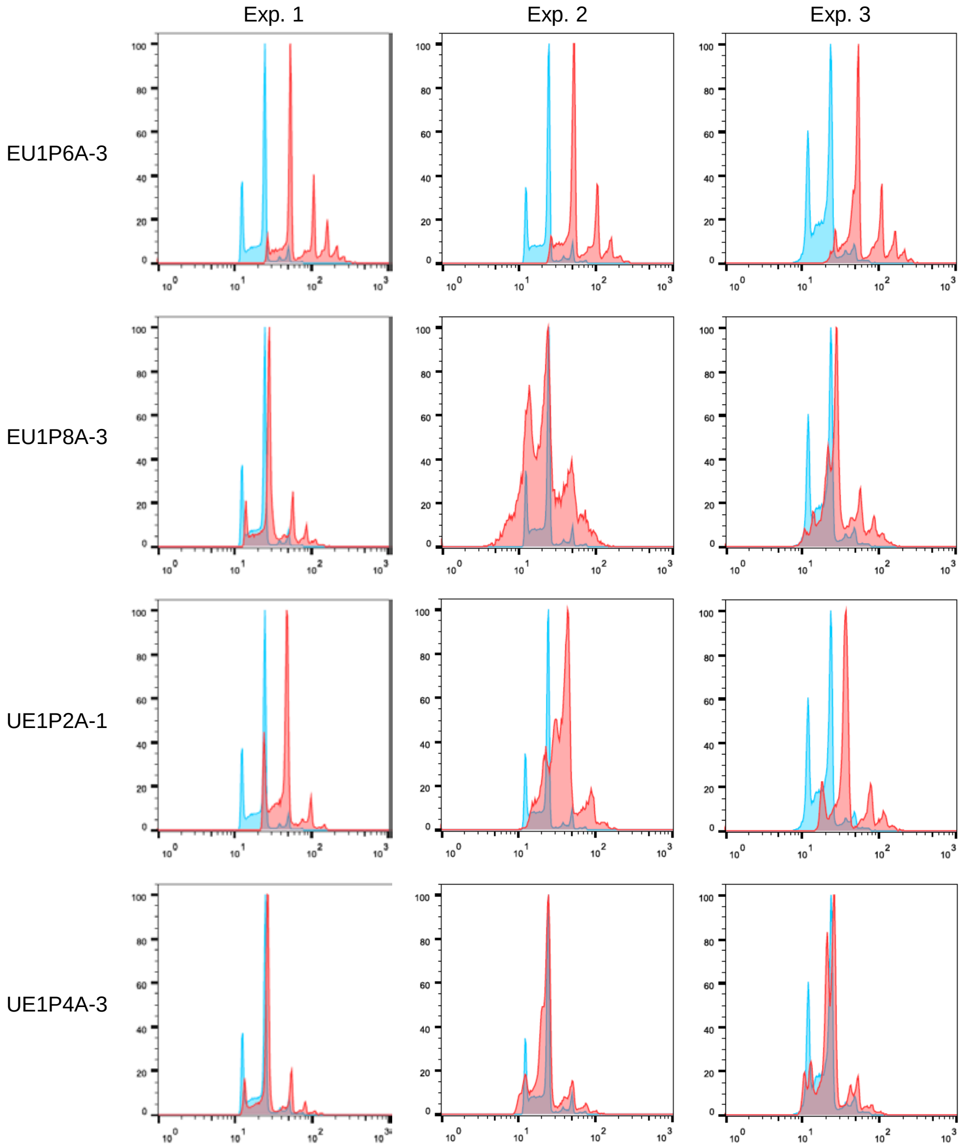

Fig S8 continued.

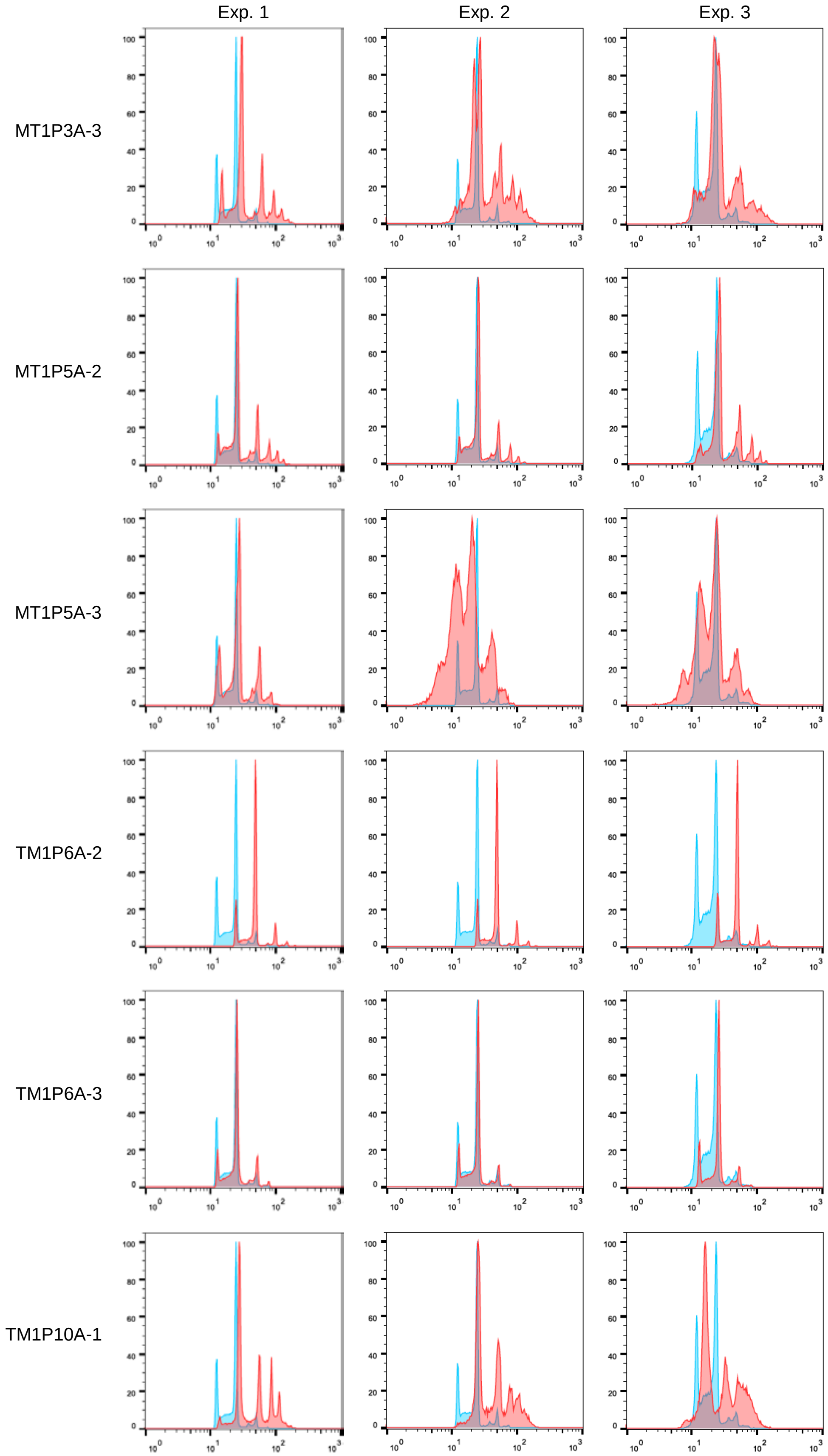

Fig S8 continued.

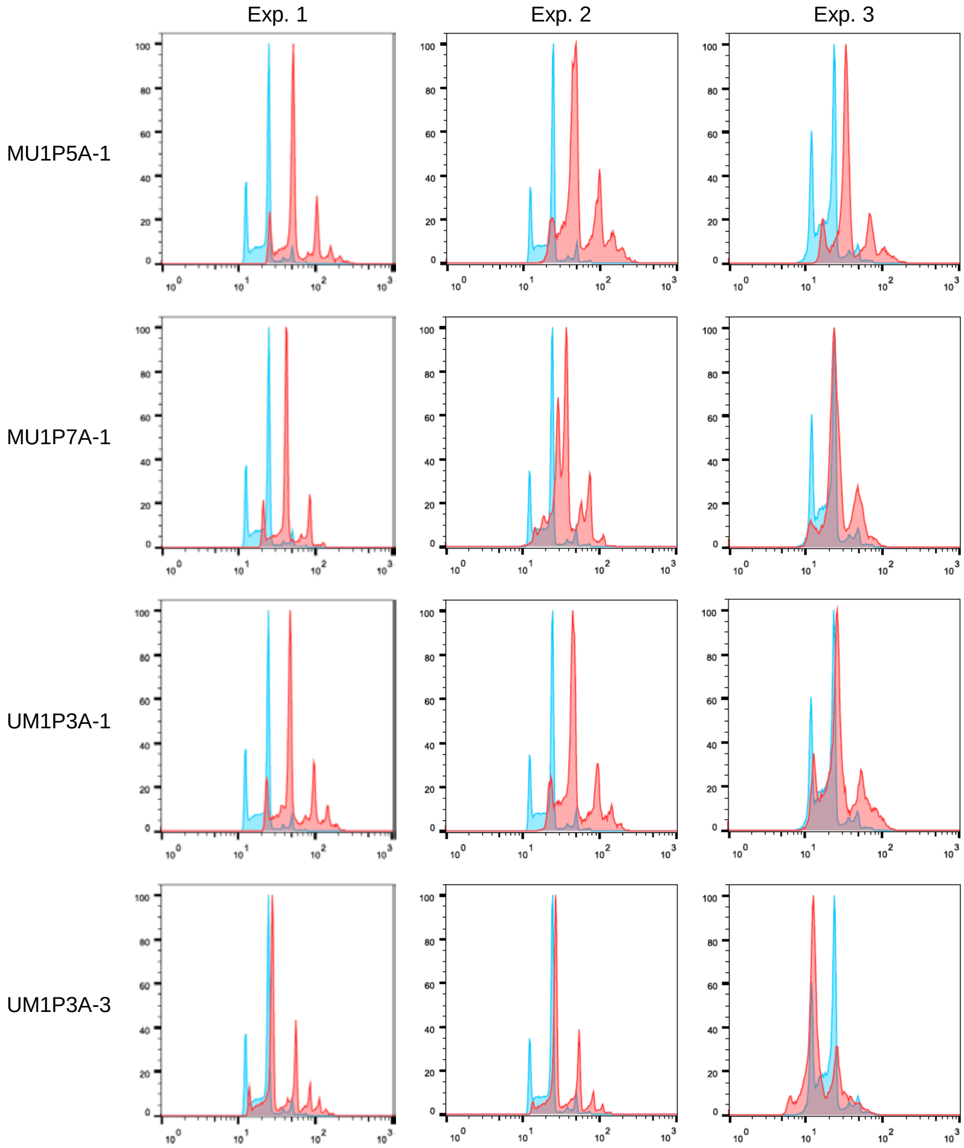

Fig S8 continued.

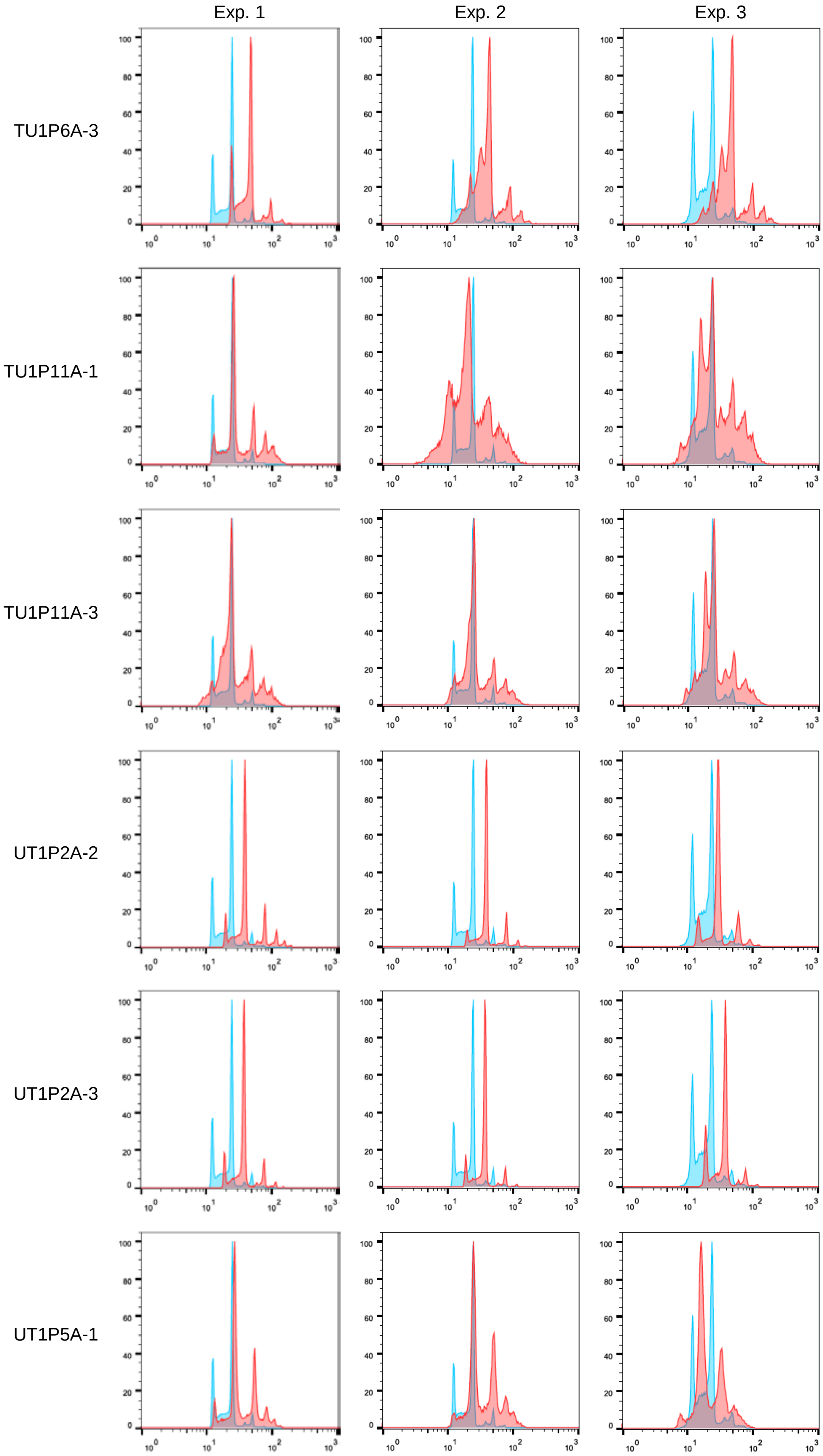

Fig S8 continued.

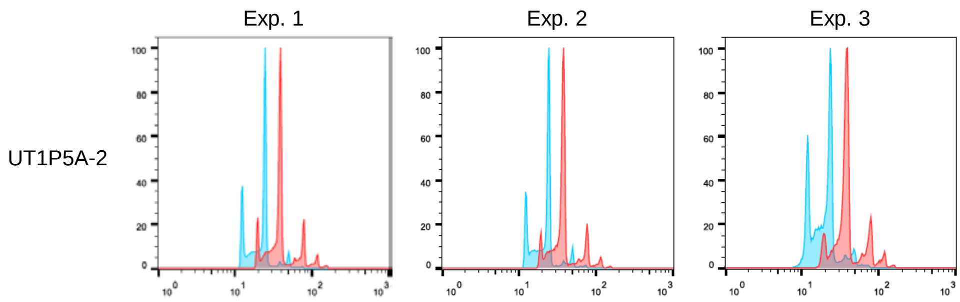

Fig S8 continued.
